# Flyway structure in the circumpolar greater white‐fronted goose

**DOI:** 10.1002/ece3.4345

**Published:** 2018-07-30

**Authors:** Robert E. Wilson, Craig R. Ely, Sandra L. Talbot

**Affiliations:** ^1^ Alaska Science Center U. S. Geological Survey Anchorage Alaska; ^2^ Institute of Arctic Biology University of Alaska Fairbanks Fairbanks Alaska

**Keywords:** *Anser albifrons*, gene flow, genetic structure, phylogeography

## Abstract

Dispersal and migratory behavior are influential factors in determining how genetic diversity is distributed across the landscape. In migratory species, genetic structure can be promoted via several mechanisms including fidelity to distinct migratory routes. Particularly within North America, waterfowl management units have been delineated according to distinct longitudinal migratory flyways supported by banding data and other direct evidence. The greater white‐fronted goose (*Anser albifrons*) is a migratory waterfowl species with a largely circumpolar distribution consisting of up to six subspecies roughly corresponding to phenotypic variation. We examined the rangewide population genetic structure of greater white‐fronted geese using mtDNA control region sequence data and microsatellite loci from 23 locales across North America and Eurasia. We found significant differentiation in mtDNA between sampling locales with flyway delineation explaining a significant portion of the observed genetic variation (~12%). This is concordant with band recovery data which shows little interflyway or intercontinental movements. However, microsatellite loci revealed little genetic structure suggesting a panmictic population across most of the Arctic. As with many high‐latitude species, Beringia appears to have played a role in the diversification of this species. A common Beringian origin of North America and Asian populations and a recent divergence could at least partly explain the general lack of structure at nuclear markers. Further, our results do not provide strong support for the various taxonomic proposals for this species except for supporting the distinctness of two isolated breeding populations within Cook Inlet, Alaska (*A. a. elgasi*) and Greenland (*A. a. flavirostris*), consistent with their subspecies status.

## 1 INTRODUCTION

Migration and dispersal play important roles in shaping both genetic and demographic structure of species (Liedvogel, Akesson, & Bensch, 2011; Moussy et al., 2013; Rolshausen, Segelbacher, Hermes, Hobson, & Schaefer, 2013). In particular, the strength of migratory connectivity within and between flyways can profoundly impact the distribution of genetic variation across a species’ range as the magnitude (degree) of connectivity can facilitate or impede genetic exchange across the landscape (Carroll et al., 2015; Ruegg et al., 2014). In general, members of a particular population or aggregation follow traditional or historical migration routes; however, individual migratory strategies can be flexible and, in some instances, can alter rapidly (Jonker et al., 2013; Pulido, 2007; Rolshausen et al., 2013; Sutherland, 1998). How this flexibility influences the genetic composition of populations will largely depend not only on the relative frequency of flyway switching (abmigration) but ultimately if these observed migratory irregularities lead to homogenization of previously separated populations (Rockwell & Barrowclough, 1987).

In northern high latitudes, most waterfowl species are highly mobile and migrate seasonally from nesting areas at higher latitudes during the summer months to areas at lower latitudes during winter months. Banding, telemetry, bird counts throughout the year, and morphological data have led to the identification of major migratory flyways, which are an integral part of management strategies, particularly in North America. However, the boundaries between these flyways are not always discrete and fidelity to these migratory flyways varies within and across taxonomic groups (Baldassarre, 2014; Ely & Scribner, 1994; Guillemain, Sadoul, & Simon, 2005; Lavretsky, Miller, Bahn, & Peters, 2014; Madsen, Tjørnløv, Frederiksen, Mitchell, & Sigfússon, 2014). Although observational data, such as the distribution of band recoveries, frequently suggest high migratory connectivity, it is relatively unknown in many waterfowl species if fidelity to migration flyway reflects philopatry (natal‐ and breeding‐site fidelity), which would promote genetic structure. Contrasting patterns in structure ascertained from genetic information and observational data have been uncovered for many avian species (e.g., Koenig, van Vuren, & Hooge, 1996; Kraus et al., 2014; Liu, Keller, & Heckel, 2012; Pearce et al., 2014), such that although observational data revealed little or no interchange, genetic data showed limited (or no) genetic signal of flyway structure. Lack of correspondence between genetic structure and observational data has been attributed to mainly male‐biased dispersal in cases where annual pair formation occurs on the winter grounds, and this has provided a mechanism enabling genetic interchange among breeding locales (see, e.g., Peters & Omland, 2007; Wilson, Gust, Petersen,  & Talbot, 2016). Waterfowl are harvested largely in wintering areas, yet population counts (upon which management decisions are based) are typically conducted on breeding areas. Thus, an understanding of the strength of the relationship between philopatry and fidelity to flyway is integral for species management and maintaining the future viability of populations (Baldassarre, 2014).

**Figure 1:**
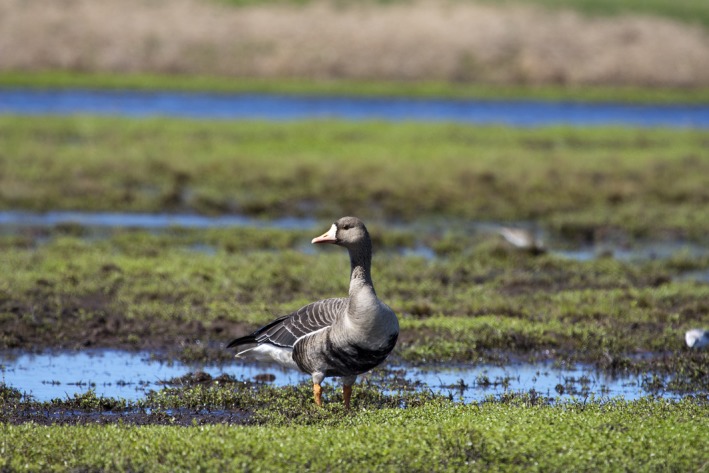
Greater white‐fronted goose in James Campbell National Wildlife Refuge, O’ahu, Hawaii, USA. Photograph credit: Robert Wilson (USGS)

**Figure 2:**
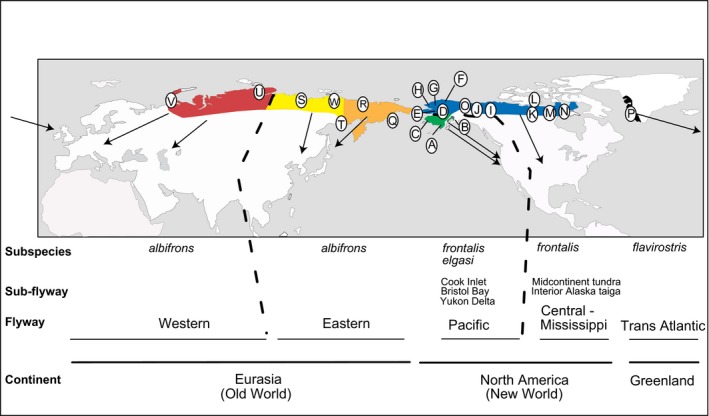
Sampling localities (A–W) of greater white‐fronted goose used in this study (refer to Table 1 for location names). The species’ breeding range is highlighted with shaded color corresponding to flyway designation and haplotypes presented in Figure 3. Suture zones at Lena River, Russia, and Mackenzie River Delta, Canada, are indicated by the letter S and J, respectively. These two locations also indicate the proposed western and eastern boundary of Beringia near the Khatanga River

Geese occupying northern high latitudes exhibit life‐history traits that may facilitate population structure and restrict interflyway genetic exchange, such as high philopatry in both sexes along with long‐term pair bonds and familial associations and delayed reproduction (Ely & Scribner, 1994; Scribner et al., 2003; Ely, Wilson, & Talbot, 2017). In contrast to ducks, pair‐bonding in some goose species is thought to occur primarily during the spring and summer when genetically similar individuals are segregated (Ely & Scribner, 1994; Leafloor, Moore, & Scribner, 2013; Weegman et al., 2015), and this would provide an additional mechanism to further limit gene flow among breeding areas. The greater white‐fronted goose (*Anser albifrons,* Figure 1) is only one of two goose species with a nearly circumpolar distribution (the other being brant, *Branta bernicla*), and is comprised of populations that utilize five major flyways (Figure 2). Across Eurasia and North America, there is considerable phenotypic variation (Ely et al., 2005), which has led to the naming of up to six morphological subspecies (Banks, 2011; Delacour, 1954; Mooij & Zöckler, 2000); Mooij and Zöckler (2000) also include an ecological component to their subspecies attributions. However, geographic distribution of some subspecies remains uncertain, and it is unclear whether there is correspondence between subspecies designations and genetic partitioning. The maintenance of subspecies boundaries and lack of interflyway banding recoveries suggest restricted gene flow among subspecies that loosely corresponds to flyway (see Ely  & Dzubin, 1994 for summary). However, distinct phenotypic variants occur in sympatry at certain periods during the annual cycle. Within the Pacific Flyway, for example, three morphologically distinct populations, including the largest and smallest forms, overlap in migratory pathways and winter distribution (Ely & Takekawa, 1996; Ely et al., 2005; Orthmeyer, Takekawa, Ely, Wege, & Newton, 1995). Several mechanisms have been proposed in the maintenance of reproductive isolation of sympatric wintering populations, such as microgeographic and behavioral barriers (see Ely et al., 2017). Furthermore, greater white‐fronted goose populations, at least in North America, tend to be spatially and temporally segregated during migration (Ely, Neiman, Alisauskas, Schmutz, & Hines, 2013; Ely & Takekawa, 1996). Further, diverse topography, as characterized by mountains and river drainages, promote temporal variation in timing of breeding and hence fall migration for breeding chronology. This is particularly evident within Alaskan breeding populations, which exhibit greater latitudinal variation in breeding chronology than other regions (Ely et al., 2005) that may promote structure both across and within flyways.

Previous population genetic studies on greater white‐fronted geese have focused on either within flyway (Ely et al., 2017; Volkovsky, Fisenko, Gerasimov, & Zhuravlev, 2016; Volkovsky, Kulikova, Gerasimov, & Zhuravlev, 2013) or between Eurasia and Greenland populations (Eda, Shimada, Ushiyama, Mizota, & Koike, 2013). Here we present the most comprehensive population genetic assessment of the greater white‐fronted goose to date across their entire breeding range, using genotypic data from eight autosomal microsatellite loci and sequence data from the mitochondrial DNA (mtDNA) control region, as well as contemporary observational data in the form of band recovery distribution through 2015. Specifically, we determined the level of genetic partitioning (continent, flyway, or within flyway) and used a Bayesian model of isolation with migration to test for the presence of intercontinental gene flow. As greater white‐fronted geese exhibit high levels of migratory connectivity (>50% return rates to both winter and breeding grounds; Fox & Stroud, 1988; Wilson, Norriss, Walsh, Fox, & Stroud, 1991; Ely & Dzubin, 1994; Alisauskas & Lindberg, 2002) and have complex long‐standing family structure, we predict there would be significant genetic structuring not only among flyways but also among breeding areas.

## 2 METHODS

### 2.1 Sample collection

Blood, feather, muscle tissue, or eggshell membranes were collected from greater white‐fronted geese throughout most of their known breeding range, including North America (eight localities representing two major flyways and two to three subspecies in Alaska and seven localities representing midcontinent breeding areas in Canada), Asia (seven localities representing 1–2 subspecies) and Greenland (one subspecies; Figure 2).

### 2.2 Study species

Within North America, the Pacific Flyway population of greater white‐fronted geese is comprised of three distinct breeding regions representing two subspecies (Figure 2): Cook Inlet (tule goose, *A. a. elagsi*; also known as *A. a. gambeli* or *A. a. gambelli*; see Banks, 2011 for summary of taxonomic history) and Yukon‐Kuskokwim Delta and Bristol Bay region (*A. a. frontalis*). The two western Alaskan breeding locales have been recently proposed but not currently accepted as comprising a separate subspecies due to their small body size (*A. a. sponsa*; Banks, 2011) with the Bristol Bay population being of intermediate body size (Orthmeyer et al., 1995). The midcontinent breeding population is located across the taiga and arctic habitats of central and northern Alaska and Canada with migratory routes mainly within the Central Flyway but to a lesser extent in the Mississippi Flyway (Figure 2). The midcontinent population is most often considered a single subspecies (*A. a. frontalis*).

Within Eurasia, the Khatanga River has been proposed as the geographic break between western and eastern Palearctic populations (Mooij & Zöckler, 2000); however, Delacour (1954) proposed this biogeographic break was further east, at the Kolyma River. Within western Beringia, the Lena River and Yana River are of particular interest in that these areas tend to have high biodiversity given they are situated in between Atlantic and Pacific flyways (Gilg et al., 2000). Although, this region is often grouped within the eastern Palearctic, results from a recent movement study (Li, Si, Ji, & Gong, 2017) showed these populations winter farther inland in China and possibly represent a more central Palearctic Flyway as Far East populations (e.g., Anadyr and Kolyma) tend to winter primarily in coastal regions of Japan and Korea. However, in agreement with Delacour (1954), observations of migrating geese suggested that the Lena River area might be partially composed of western Palearctic geese (Syroechkovskiy, 2006). Thus, the subspecific designation of geese in these regions, which include much of western Beringia, is still in debate. While some authorities consider that all Eurasian populations comprise a single subspecies (*A. a. albifrons*; Owen, 1980; Portenko, 1989; Banks, 2011), others (e.g., Delacour, 1954) place the eastern Palearctic populations within the North American subspecies (*A. a. frontalis*), based on similarity in body size. Mooji (2000) and Mooij and Zöckler (2000) further propose designating the eastern Palearctic population as a separate subspecies (*A. a. albicans*), based on wintering distribution, migratory routes, and slightly larger body size than western Palearctic areas. Geese nesting in Greenland and wintering primarily in Ireland and United Kingdom are designated as a separate subspecies (*A. a. flavirostris*) due to their large size, dark coloration and nonoverlapping breeding and wintering distribution with other potential subspecies.

### 2.3 DNA isolation and sequencing

Genomic DNA was extracted from blood, muscle, feather, or eggshell membranes using a “salting out” procedure described by Medrano, Aasen, and Sharrow (1990), with modifications described in Sonsthagen, Talbot, and White (2004) for blood and muscle and in Talbot et al. (2011) for feathers and eggshell membranes. Genomic DNA concentrations were quantified using fluorometry and diluted to 50 ng mL^–1^ working solutions.

We amplified a portion of domain I and II of the mtDNA control region using the primer pair WFGL1M (5′–ACTAACCGCGAACTCCCAAA–3′) and H542 (Sorenson & Fleischer, 1996), yielding a 366‐bp fragment for all individuals. PCR amplifications, cycle‐sequencing protocols, and postsequencing processing followed Sonsthagen et al. (2004).

Initially, 12 individuals were screened for variability at 26 loci known to be variable in other waterfowl species. Eight unlinked polymorphic loci with dinucleotide repeat motifs and in Hardy–Weinberg equilibrium were selected for further analysis; BCA5, BCA6, BCA9, BCA11 (Buchholz, Pearce, Pierson, & Scribner, 1998), CRG (Wilson et al., 2016), OXY13 (Muñoz‐Fuentes, Gyllenstrand, Negro, Green, & Vila, 2005), TSP1.20.09, and TSP1.20.46 (St. John, Ransler, Quinn, & Oyler‐McCance, 2006). Linkage disequilibrium (LD) for each locus and population was calculated in FSTAT ver. 2.9.3 (Goudet, 1995) and Hardy–Weinberg equilibrium (HWE) in Genepop (Raymond & Rousset, 1995; Rousset, 2008). Polymerase chain reaction (PCR) amplification and electrophoresis followed protocols described in Sonsthagen et al. (2004). Ten percent of the samples were amplified and genotyped in duplicate for the eight microsatellite loci for quality control purposes.

### 2.4 Genetic diversity

We calculated basic population genetic parameters, haplotype (*h*) and nucleotide (*π*) diversity, for mtDNA control region using ARLEQUIN ver. 3.5.1.2 (Excoffier & Lischer, 2010). In addition, an unrooted phylogenetic tree for mtDNA control region was constructed in NETWORK 4.6.1.3 (Fluxus Technology Ltd., 2009) using the median joining network method Bandelt, Forster, and Röhl (1999), to illustrate possible reticulations in the gene tree because of homoplasy. For microsatellites, allelic richness, observed and expected heterozygosities were calculated in FSTAT ver. 2.9.3 (Goudet, 1995).

### 2.5 Population subdivision

The degree of population subdivision among breeding areas was assessed by calculating pairwise *F*_ST_ for mtDNA and microsatellite in ARLEQUIN, adjusting for multiple comparisons using Benjamini and Yekutieli‐modified false discovery rate (*α* = 0.05; Benjamini & Yekutieli, 2001; Narum, 2006). Because the upper possible *F*_ST_ value for a set of microsatellite loci is usually <1.0 (Hedrick & Goodnight, 2005), we used RECODEDATA, version 1.0 (Meirmans, 2006), to calculate the uppermost limit of *F*_ST_ for a given data set.

We used two approaches to explore the genetic partitioning of genetic variation among and within breeding groups. We first used an analysis of molecular variance (AMOVA) in ARLEQUIN to test for significance of geographic partitioning of a priori hypothesized genetic units using mtDNA and microsatellite loci with statistical significance tested by 16,000 permutations. Populations were grouped to test (see Figure 1): (a) current and prior subspecific groupings, (b) geographic/flyway division, (c) nesting habitat—tundra vs. taiga and (d) proposed refugia (see Ploeger, 1968). As Greenland and Cook Inlet both only represented a single subspecies, we excluded these populations from subspecies groupings, as having a group represented by a single population will bias within‐ and between‐group variance. As well, Greenland was excluded from major flyway groupings, as it is the only population utilizing its flyway. We assumed that groupings that maximize among‐group variation (Φ_CT_ or *F*_CT_) and significantly different than random distribution of individuals were the most probable geographic divisions (*p *<* *0.05).

Secondly, we used a Bayesian‐clustering program, STRUCTURE 2.2.3 (Pritchard, Stephens, & Donnelly, 2000), to determine the level of population structure in the autosomal microsatellite data set without providing a priori information on the geographic origin of the individuals. If no structure was observed, the LOCPRIOR option was used as this model is able to detect population structure in datasets with a weak signal of structure not detectable under standard models (Hubisz, Falush, Stephens, & Pritchard, 2009). STRUCTURE assigns individuals to populations maximizing Hardy–Weinberg equilibrium and minimizing linkage disequilibrium. The analysis was run for *K *=* *1–20, where *K* is the number of populations, using an admixture model with 100,000 burn‐in iterations and 1,000,000 Markov chain Monte Carlo (MCMC) iterations. Initially the analyses were repeated five times for each *K* and based on these preliminary results, 10 additional replicated were performed for *K *=* *1–10 for a total of fifteen independent runs. We used the ∆*K* method of Evanno, Regnaut, and Goudet (2005) and evaluated the estimate of the posterior probability of the data given *K*, Ln *P*(*D*), to determine the most likely number of groups at the uppermost level of population structure.

### 2.6 Demographic history and gene flow

Demographic histories of the greater white‐fronted goose based on mtDNA sequence data were evaluated using two approaches: standard qualitative test statistics, Tajima's *D* and Fu's *F*_s_, and coalescent‐based estimation implemented in IMa2 (Hey & Nielsen, 2004). To test for genetic signatures of recent effective population size changes, we calculated Fu's *F*_s_ (Fu, 1997) and Tajima's *D* (Tajima, 1989) on the basis of the site‐frequency spectrum of segregating sites with statistical significance evaluated by 16,000 simulated samples. Negative values of Tajima's *D* or Fu's *F*_s_ result when there is an excess of low‐frequency polymorphisms, which can result from rapid population expansion or selective sweep acting on linked polymorphisms. Conversely, a positive value for either test statistic can be indicative of a population decline.

To estimate levels of gene flow, we used the Isolation with Migration model, IMa2. To define a population tree, we estimated the phylogenetic relationships among populations in *BEAST version 1.8.2 (Heled & Drummond, 2010). *BEAST uses Bayesian analysis incorporating a Markov Chain Monte Carlo (MCMC) in phylogeny estimation (Heled & Drummond, 2010). We ran 50,000,000 iterations, sampling every 2,000 MCMC steps following a burn‐in of 5,000,000 steps using a strict clock model. To obtain an averaged tree with posterior distributions we used TreeAnnotator version 1.7 (Drummond, Suchard, Xie, & Rambaut, 2012) removing the first 2,500 trees as burn‐in. Due to the uncertainty of the divergence events among populations, evident by low posterior distributions of branches (<50), we simplified the gene flow model by only assessing gene flow among continents (Old vs. New World populations; see Table 1 for population groupings) as the breeding locales generally grouped by continent; albeit with low support (Supporting Information Appendix S1). Preliminary results using different evolutionary divergence scenarios between major flyways yielded differing rates and directionality of gene flow, further suggesting that the simplified model of Old vs. New World was most appropriate given the dataset.

**Table 1:** Sampling localities and genetic diversity measures^a^

Land Mass^b^	Flyway^c^	Region	Locality (Map code^d^)	mtDNA						Microsatellites			
				*n*	*H*	dh (*SD*)	*π* (*SD*)	D	*F*	*n*	*A* (*SD*)	AR	Ho (*SD*)/He (*SD*)
OW	Western Palearctic	Greenland	Ireland (P)	29	12	0.9039 (0.0305)	0.0084 (0.0050)	−0.57	−3.29	30	4.00 (1.77)	2.83	54.6 (3.2)/51.8 (5.9)
		Asia	Vaygach Island (V)	17	5	0.7132 (0.0827)	0.0093 (0.0056)	−0.04	2.42	17	5.00 (2.88)	3.65	61.2 (4.2)/61.2 (8.0)
			Taimyr Peninsula (U)	20	12	0.9211 (0.0422)	0.0154 (0.0086)	−0.96	−1.73	20	5.38 (2.26)	3.60	61.7 (3.9)/60.8 (6.2)
	Eastern Palearctic	Asia	Lena River Delta (S)	10	6	0.7778 (0.1374)	0.0047 (0.0034)	−0.84	−2.29	10	4.13 (2.70)	3.48	51.3 (5.6)/60.9 (7.1)
			Yana River Delta (W)	13	6	0.6410 (0.1498)	0.0048 (0.0033)	−**2.11**	−1.59	15	4.25 (2.25)	3.43	58.0 (4.6)/63.2 (6.0)
			Magadan (T)	5	4	0.9000 (0.1610)	0.0109 (0.0077)	−0.86	0.05	5	3.38 (1.69)	3.38	60.0 (7.8)/58.1(8.6)
			Kolyma River Delta (R)	22	18	0.9827 (0.0183)	0.0159 (0.0088)	−0.02	−**9.62**	23	5.25 (3.01)	3.66	64.7 (3.5)/64.8(5.2)
			Anadyr Lowlands (Q)	25	13	0.9233 (0.0300)	0.0193 (0.0104)	1.35	−1.18	34	5.38 (3.20)	3.40	52.6 (3.0)/59.3(7.2)
NW	Midcontinent	Alaska	Selawik NWR (G)	36	25	0.9698 (0.0157)	0.0205 (0.0109)	−0.12	−**10.99**	40	5.25 (3.01)	3.47	61.9 (2.7)/61.6 (6.1)
			Koyukuk NWR (E)	49	14	0.8189 (0.0406)	0.0182 (0.0097)	0.52	0.36	39	5.63 (2.92)	3.58	66.8 (2.7)/63.4 (5.8)
			Kanuti NWR (D)	25	13	0.8667 (0.0607)	0.0213 (0.0114)	0.86	−0.91	30	5.38 (2.20)	3.68	62.8 (3.1)/63.3 (7.0)
			Point Lay (H)	26	4	0.4431 (0.1039)	0.0014 (0.0014)	−0.03	−1.25	31	4.75 (2.49)	3.49	63.9 (3.1)/62.7 (6.4)
			North Slope (F)	31	13	0.8860 (0.0391)	0.0141 (0.0078)	−0.31	−1.46	64	6.13 (3.98)	3.60	60.9 (2.2)/62.1(6.1)
		Canada	Old Crow, Yukon (O)	22	12	0.8658 (0.0652)	0.0186 (0.0102)	0.39	−1.09	28	5.00 (2.45)	3.48	59.8 (3.3)/61.7 (7.0)
			Anderson River (I)	23	13	0.9091 (0.0423)	0.0170 (0.0094)	−0.07	−2.14	25	5.25 (2.82)	3.73	60.5 (3.5)/65.6 (5.4)
			Mackenzie River, NWT (J)	15	8	0.8667 (0.0673)	0.0150 (0.0086)	0.47	−0.09	17	4.88 (2.42)	3.45	57.4 (4.2)/62.3(5.2)
			Kent Peninsula (K)	20	8	0.7421 (0.0961)	0.0089 (0.0053)	−1.19	−0.77	23	4.75 (2.31)	3.49	61.4 (3.6)/62.2 (6.4)
			Victoria Island (L)	28	15	0.8836 (0.0496)	0.0180 (0.0098)	1.71	−2.64	34	5.38 (3.07)	3.33	57.7 (3.0)/58.6 (7.9)
			Queen Maud Gulf (M)	24	14	0.9493 (0.0243)	0.0134 (0.0076)	0.09	−4.06	34	5.38 (3.20)	3.56	58.8 (3.0)/61.1(7.0)
			Rasmussen Basin (N)	24	11	0.9130 (0.0308)	0.0132 (0.0074)	0.02	−1.32	29	5.25 (3.24)	3.27	56.5 (3.3)/57.8 (6.3)
	Pacific	Alaska	Yukon‐Kuskokwim Delta (C)	27	17	0.9573 (0.0210)	0.0217 (0.0116)	0.85	−3.42	36	5.50 (3.51)	3.62	58.3 (2.9)/59.8 (8.3)
			Bristol Bay (A)	19	8	0.8304 (0.0657)	0.0109 (0.0064)	0.02	−0.30	20	4.50 (2.88)	3.38	57.6 (4.0)/59.4(7.0)
			Cook Inlet (B)	40	12	0.7679 (0.0600)	0.0094 (0.0054)	−0.58	−1.61	61	5.00 (2.33)	3.28	60.1 (2.2)/60.0(5.7)

^a^ *n* = sample size, *H* = number of haplotypes, dh = haplotype diversity, *π* = nucleotide diversity, *D *= Tajima's *D* (significant values in bold), *F *= Fu's *F*_S_ (significant values in bold), *A* = average number of alleles per locus, AR = allelic richness based on sample size of 5, Ho = percent observed heterozygosity, and He = percent expected heterozygosity. ^b^Land masses are defined as Old World (OW, Eurasia) and New World (NW, North America). Greenland breeding population is included in Old World because this population winters in Europe. ^c^Eastern and western Palearctic designations follow Mooij & Zöckler, 2000;. ^d^Map code refers to Figure 1.

Here, we estimated three different evolutionary parameters including ϴ (where ϴ = 4*N*_e_ *μ*, and *N*_e_ is the effective population size and *μ* is the mutation rate per site per generation) for each contemporary population and the ancestral populations, 2*Nm* (which is *ϴm*_im_/2, here *m*_im_ is the migration rate relative to the mutation rate estimated in IMa2, *N* is the population size, and *m* is the proportion of the population consisting of immigrants per generation), and *t* (where *t *= *T*/*μ*, and *T* is the number of years since divergence). We ran 20 Markov chains under a geometric heating scheme (option ‐hfg), with chain temperatures of 0.90 and 0.75. We ran preliminary analyses using large, flat priors for each parameter. From the results of these runs, we defined narrower upper bounds for each parameter that encompassed the full posterior distributions from each initial run. The final values used for population size, migration rate and splitting time were as follows: −q 300, −m 10, −t 5. The analysis was repeated four times to check for convergence, and all ESS values were >100 in all runs. To convert the estimated parameters into demographic units we used a mutation rate of 4.8 × 10^−8^ (confidence interval 3.1–6.9 × 10^−8^) substitutions per site per year that has been previously used for other waterfowl studies (Kraus et al., 2011; Peters, Gretes, & Omland, 2005; Wilson, Peters, & McCracken, 2013; Wilson et al., 2011). We calculated generation time (*G*) using the equation *G* = *α* + (*s*/(1 − s)), where *α* is the age of maturity and s is the expected adult survival rate (Sæther et al., 2005). Greater white‐fronted geese reach sexual maturity at age 3 (Baldassarre, 2014; Campbell & Goodwin, 1985), and adult survival rates averaged 0.749 of the Pacific flyway population (Schmutz & Ely, 1999). Using those values, we estimated *G* to be approximately 5.9 years, which corresponds to generation time estimates of other goose species (Dillingham, 2010).

### 2.7 Compilation of band recovery data

We obtained banding and recovery data from the U. S. Geological Survey (USGS) Bird Banding Laboratory (BBL) in Laurel, Maryland for greater white‐fronted geese which included data from birds banded or recovered in North America through 2015. To assess intercontinental dispersal and flyway switching within Eurasia, we contacted the BBL directly (D. Bystrak, pers comm. December 29, 2015), the Russian Ringing Centre (K. Litvin, pers. com.), and accessed Euring data (T. Fox, pers. com.). Banding records came from geese captured on breeding and wintering areas, but there were very few recoveries (or observations) on northern breeding areas. More information on band recovery data is available in Supporting Information Appendix S2.

## 3 RESULTS

### 3.1 Genetic diversity

**Figure 3:**
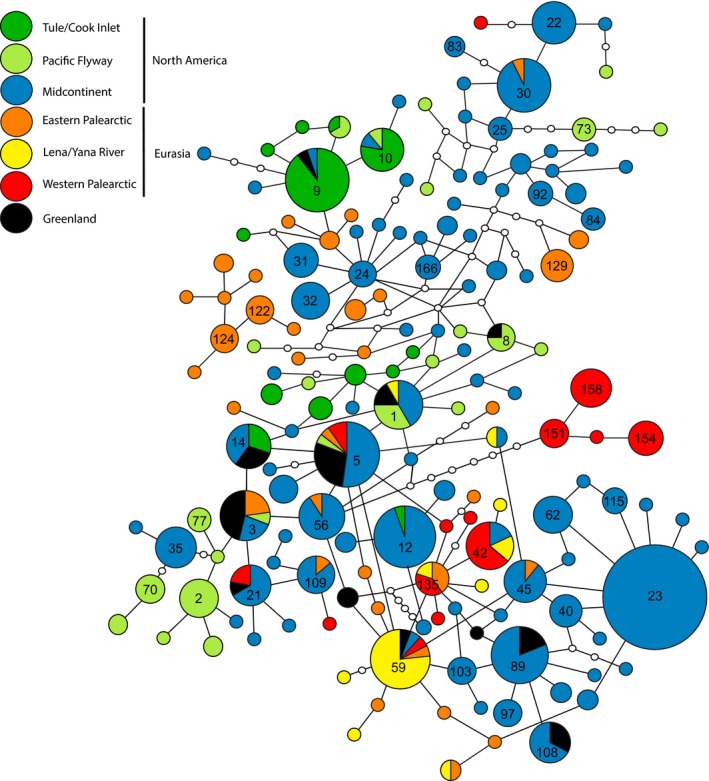
Unrooted haplotype network for greater white‐fronted geese. Size of circles is proportional to the frequency of each haplotype observed. Small white circles indicate haplotypes not observed in this study. For sampling location assignment to each group, refer to Table 1 and Figure 2. In general, Pacific Flyway is composed of population codes A (Bristol Bay) and C (Y‐K Delta) as well as the Tule Goose/Cook Inlet (population code B). The Midcontinent population is represented by samples from Alaska (D–H) and Canada (I–N). Western Palearctic consists of Taimyr (U) and Vaygach Island (V). Eastern Palearctic is represented by Anadyr Lowlands (Q), Kolyma River Delta (R) and Magadan (T). Although considered apart of eastern Palearctic, Lena River (S) and Yana River (W) are indicated in yellow as these locales represent a potential transition from western to eastern Palearctic populations. Greenland population is indicated by the population code P

**Figure 4:**
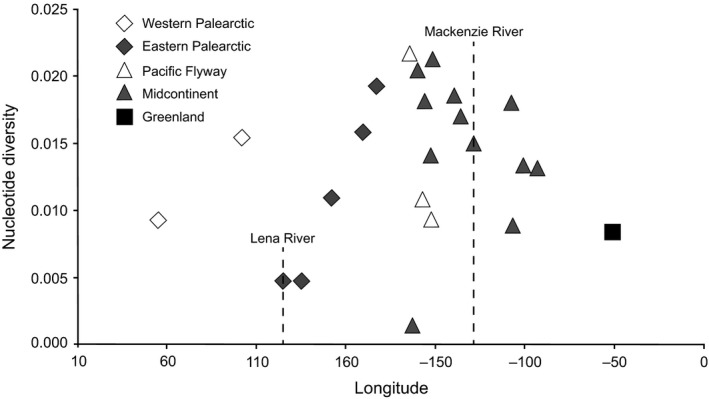
Plot of mtDNA control region nucleotide diversity (*π*) by longitude. Boundaries of Beringia as defined by Hultén (1937) are indicated by dashed line which also represent two well‐known suture zones at Lena River, Russia and Mackenzie River Delta, Canada

One hundred and sixty‐two unique mtDNA haplotypes were found from 550 individuals representing 23 different breeding localities (Figure 3). Of the 366 aligned nucleotide positions, 70 (19%) were variable and two sites had deletions. Of the 162 haplotypes, 96 haplotypes (59%) were only represented by a single individual, 55 haplotypes by 2–10 individuals, nine haplotypes by 11–20 individuals, one haplotype by 21 individuals, and the most frequent haplotype (Hap 23) was represented by 73 individuals (Figure 3, Supporting Information Appendix S3). This haplotype (23) is found in only the New World midcontinent population. Further, only 16 haplotypes (10% of all haplotypes) were shared among Old and New World populations (including Greenland), six (5% of all North American haplotypes) were shared between North American flyways, and two (4% of all Asian haplotypes) were shared between western (Taimyr and Vaygach) and eastern (Anadyr, Kolyma River, and Magadan) Palearctic populations (excluding Greenland). Within the Palearctic, Lena and Yana River areas shared three haplotypes each with eastern and western Palearctic populations. Old and New World populations had similar nucleotide diversity with the lowest values being found in the Lena River and Yana River Deltas in Central Siberia and Point Lay, Alaska (Table 1). Overall there were no significant trends in nucleotide diversity with longitude within each continent (Figure 4). However, when considering only major flyways, there was a significant decreasing west to east trend within the North American midcontinent population after the removal of Point Lay (*r* ^2^ = 0.44 *p *=* *0.025) and east to west within the eastern Palearctic Flyway (*r* ^2^ = 0.97 *p *=* *0.003).

Based on 665 individuals genotyped, the number of alleles per autosomal microsatellite locus ranged from 3 (BCA 6) to 16 (OXY 13), with an average of 8.38 (*SD* 4.14) alleles per locus (Table 1). Molecular diversity indices were similar across regions with allelic richness ranging from 2.83 (Greenland) to 3.73 (Anderson River, Canada; Table 1). Observed heterozygosity ranged from 51.3% (Lena River) to 66.8% (Koyukuk, Alaska) with an overall value of 59.9% (*SD* 6.7%). All populations and loci were in Hardy–Weinberg and linkage equilibrium. No significant correlation with allelic richness and longitude was detected within Old or New World regions or within each flyway.

### 3.2 Low microsatellite differentiation but high mtDNA differentiation

We found significant overall differentiation for mtDNA (*F*_ST_ = 0.131, Φ_ST_ = 0.295, *p *<* *0.05). However, not all sampling sites were significantly differentiated from each other (Table 2). Notably the few nonsignificant pairwise *F*_ST_ comparisons were primarily restricted to either between Palearctic populations or within the North American midcontinent. In the case of the Magadan population, sample size was low (*n *=* *5) so nonsignificant pairwise *F*_ST_ values associated with Magadan may reflect Type II error.

**Table 2:**
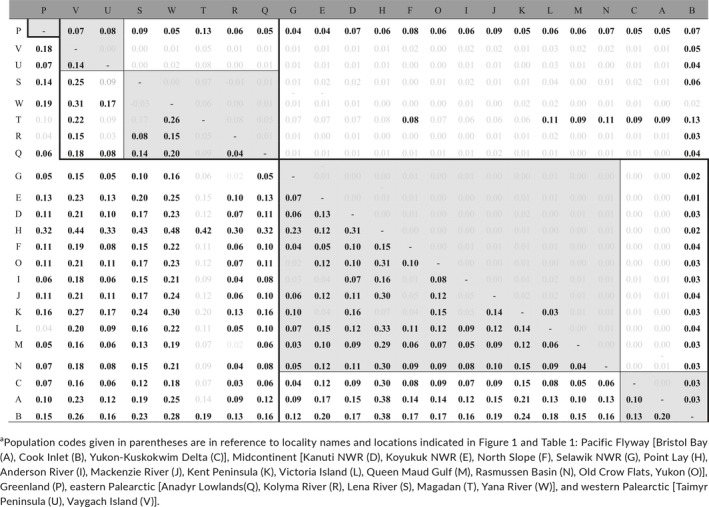
Pairwise *F*_ST_ values for microsatellite data (above diagonal) and mtDNA control region (below diagonal). *F*_ST_ values in bold text are significant after Benjamini and Yekutieli‐modified false discovery rate correction. Values outlined in thick lines include within continent comparisons and thin line and shaded comparisons indicate are within flyway comparisons. Eastern and western Palearctic Flyway designations follow Mooij and Zöckler (2000). Sampling locations are coded as letters (A–W) and refer to codes given in Table 1 and Figure 2 ^a^

An analysis of variance (AMOVA) revealed that the best partition of genetic variance was when all populations were grouped primarily by major flyway (western Palearctic, eastern Palearctic, Pacific, and midcontinent, Table 3) with Lena River and Yana River representing a potential central Palearctic Flyway (Φ_CT_ = 0.117, *p *=* *0.001). Alternatively, if Lena River and Yana River was placed within the eastern or western Palearctic, 9.3% and 10.8% of the genetic variation was explained with these groupings (Table 3). Most geographic proximity groupings with the inclusion of Greenland also explained a significant portion of variance (5.5%–11.9%; Table 3 and Supporting Information Appendix S4). The best geographic proximity grouping (11.9% of genetic variation explained; Φ_CT_ = 0.119, *p *=* *0.001) was the same as best flyway grouping with the only difference was Greenland was included within North American midcontinent population. In general, the inclusion of Greenland within the western Palearctic for hypothesized geographic groupings generally lowered the amount of genetic variance explained compared to when Greenland was placed within North America (Supporting Information Appendix S4). Groupings based on nesting habitat did not explain a significant amount of generic variation while groupings based on proposed refugia (Ploeger, 1968) explained 5.2% of the variance (Table 3).

**Table 3:** Hierarchical analysis of molecular variance of haplotypic and allelic frequencies to test hypotheses associated with (a) subspecies classification schemes; (b) flyway designation; (c) geographic proximity; (d) nesting habitat; (e) putative refugia for greater white‐fronted goose populations. For complete list of hypothesized groupings, see Supporting Information Appendix S4. Some populations were not included in all groupings as it would be the sole representative for that group. For example, Cook Inlet and Greenland populations were excluded from subspecies grouping analysis as they are the only members of their respective subspecies. Significant fixation indices (*p *<* *0.05) are indicated in bold. Please refer to Figure 1 and Table 1 for geographic location of each population name^a^

Model	Hypothesized groupings	Variance components				
		Φ_ST_	Φ_SC_	Φ_CT_	% Among groups	*p*_(among group)_
*MtDNA Control Region*						
Subspecies	[Bristol Bay, YKD, Point Lay, North Slope, Koyukuk, Kanuti, Selawik, Yukon, Anderson, Mackenzie, Kent, Queen Maud, Victoria, Rasmussen, Kolyma, Anadyr, Magadan, Yana, Lena] [Taimyr, Vaygach]	**0.357**	**0.257**	0.135	13.5	0.059
Subspecies	[Bristol Bay, YKD, Point Lay, North Slope, Koyukuk, Kanuti, Selawik, Yukon, Anderson, Mackenzie, Kent, Queen Maud, Victoria, Rasmussen, Kolyma, Anadyr, Magadan, Yana] [Taimyr, Vaygach, Lena]	**0.333**	**0.259**	**0.099**	9.9	0.027
Flyway	[Vaygach, Taimyr] [Lena, Yana, Kolyma, Magadan, Anadyr] [Point Lay, North Slope, Koyukuk, Kanuti, Selawik, Yukon, Anderson, Mackenzie, Kent, Queen Maud, Victoria, Rasmussen] [YKD, Bristol Bay, Cook Inlet]	**0.322**	**0.256**	**0.093**	9.3	0.005
Flyway	[Vaygach, Taimyr, Lena, Yana] [Kolyma, Magadan, Anadyr] [Point Lay, North Slope, Koyukuk, Kanuti, Selawik, Yukon, Anderson, Mackenzie, Kent, Queen Maud, Victoria, Rasmussen] [YKD, Bristol Bay, Cook Inlet]	**0.327**	**0.248**	**0.108**	10.8	0.002
Flyway	[Vaygach, Taimyr] [Yana River, Lena River] [Kolyma, Magadan, Anadyr] [Point Lay, North Slope, Koyukuk, Kanuti, Selawik, Yukon, Anderson, Mackenzie, Kent, Queen Maud, Victoria, Rasmussen] [YKD, Bristol Bay, Cook Inlet]	**0.329**	**0.241**	**0.117**	11.7	0.001
Geographic	[Vaygach, Taimyr, Lena, Yana] [Kolyma, Magadan, Anadyr] [Point Lay, North Slope, Koyukuk, Kanuti, Selawik, Yukon, Anderson, Mackenzie, Kent, Queen Maud, Victoria, Rasmussen, Greenland] [YKD, Bristol Bay, Cook Inlet]	**0.328**	**0.247**	**0.108**	10.8	0.001
Geographic	[Vaygach, Taimyr] [Lena, Yana] [Kolyma, Magadan, Anadyr] [Point Lay, North Slope, Koyukuk, Kanuti, Selawik, Yukon, Anderson, Mackenzie, Kent, Queen Maud, Victoria, Rasmussen, Greenland] [YKD, Bristol Bay, Cook Inlet]	**0.331**	**0.240**	**0.119**	11.9	0.001
Geographic	[Vaygach, Taimyr, Lena, Yana] [Kolyma, Magadan, Anadyr] [Point Lay, North Slope, Koyukuk, Kanuti, Selawik, Yukon, Anderson, Mackenzie] [Kent, Queen Maud, Victoria, Rasmussen, Greenland] [YKD, Bristol Bay, Cook Inlet]	**0.309**	**0.245**	**0.085**	8.5	0.002
Refugia	[Vaygach, Taimyr, Lena, Yana] [Kolyma, Magadan, Anadyr, Point Lay, North Slope, YKD, Bristol Bay, Cook Inlet, Koyukuk, Kanuti, Selawik, Anderson, Mackenzie, Yukon] [Kent, Queen Maud, Victoria, Rasmussen]	**0.316**	**0.278**	**0.052**	5.2	0.037
Nesting habitat	[Greenland, Vaygach, Taimyr, Lena, Yana, Kolyma, Anadyr, Point Lay, North Slope, Bristol Bay, Cook Inlet, YKD, Anderson, Mackenzie, Kent, Queen Maud, Victoria, Rasmussen][Magadan, Cook Inlet, Koyukuk, Selawik, Kanuti, Yukon]	**0.304**	**0.288**	0.022	2.2	0.145

		*F*_ST_	*F*_SC_	*F*_CT_	% Among groups	*p*_(among group)_
*Microsatellite loci*						
Subspecies	[Bristol Bay, YKD, Point Lay, North Slope, Koyukuk, Kanuti, Selawik, Yukon, Anderson, Mackenzie, Kent, Queen Maud, Victoria, Rasmussen, Kolyma, Anadyr, Magadan, Yana, Lena] [Taimyr, Vaygach]	**0.012**	**0.007**	**0.006**	0.60	0.052
Subspecies	[Bristol Bay, YKD, Point Lay, North Slope, Koyukuk, Kanuti, Selawik, Yukon, Anderson, Mackenzie, Kent, Queen Maud, Victoria, Rasmussen, Kolyma, Anadyr, Magadan, Yana] [Taimyr, Vaygach, Lena]	**0.006**	**0.013**	**0.007**	0.70	0.013
Flyway	[Vaygach, Taimyr] [Lena, Yana, Kolyma, Magadan, Anadyr] [Point Lay, North Slope, Koyukuk, Kanuti, Selawik, Yukon, Anderson, Mackenzie, Kent, Queen Maud, Victoria, Rasmussen] [YKD, Bristol Bay, Cook Inlet]	**0.014**	**0.009**	**0.006**	0.60	0.009
Flyway	[Vaygach, Taimyr, Lena, Yana] [Kolyma, Magadan, Anadyr] [Point Lay, North Slope, Koyukuk, Kanuti, Selawik, Yukon, Anderson, Mackenzie, Kent, Queen Maud, Victoria, Rasmussen] [YKD, Bristol Bay, Cook Inlet]	**0.014**	**0.009**	**0.005**	0.50	0.006
Flyway	[Vaygach, Taimyr] [Kolyma, Magadan, Anadyr] [Point Lay, North Slope, Koyukuk, Kanuti, Selawik, Yukon, Anderson, Mackenzie, Kent, Queen Maud, Victoria, Rasmussen] [YKD, Bristol Bay, Cook Inlet]	**0.014**	**0.009**	**0.006**	0.60	0.018
Geographic	[Vaygach, Taimyr, Lena, Yana] [Kolyma, Magadan, Anadyr] [[Point Lay, North Slope, Koyukuk, Kanuti, Selawik, Yukon, Anderson, Mackenzie, Kent, Queen Maud, Victoria, Rasmussen, Greenland] [YKD, Bristol Bay, Cook Inlet]	**0.018**	**0.014**	0.004	0.40	0.077
Geographic	[Vaygach, Taimyr] [Lena, Yana] [Kolyma, Magadan, Anadyr] [Point Lay, North Slope, Koyukuk, Kanuti, Selawik, Yukon, Anderson, Mackenzie, Kent, Queen Maud, Victoria, Rasmussen, Greenland] [YKD, Bristol Bay, Cook Inlet]	**0.017**	**0.017**	0.001	0.10	0.365
Geographic	[Vaygach, Taimyr, Lena, Yana] [Kolyma, Magadan, Anadyr] [Point Lay, North Slope, Koyukuk, Kanuti, Selawik, Yukon, Anderson, Mackenzie] [Kent, Queen Maud, Victoria, Rasmussen, Greenland] [YKD, Bristol Bay, Cook Inlet]	**0.017**	**0.013**	**0.004**	0.40	0.015
Refugia	[Vaygach, Taimyr, Lena, Yana] [Kolyma, Magadan, Anadyr, Point Lay, North Slope, YKD, Bristol Bay, Cook Inlet, Koyukuk, Kanuti, Selawik, Anderson, Mackenzie, Yukon] [Kent, Queen Maud, Victoria, Rasmussen]	**0.011**	**0.010**	0.002	0.20	>0.05
Nesting Habitat	[Greenland, Vaygach, Taimyr, Lena, Yana, Kolyma, Anadyr, Point Lay, North Slope, Bristol Bay, Cook Inlet, YKD, Anderson, Mackenzie, Kent, Queen Maud, Victoria, Rasmussen][Magadan, Cook Inlet, Koyukuk, Selawik, Kanuti, Yukon]	**0.018**	**0.015**	0.003	0.30	>0.05

a Population codes given in parentheses are in reference to locality names and locations indicated in Figure 1 and Table 1: Bristol Bay (A), Cook Inlet (B), Yukon‐Kuskokwim Delta or YKD (C), Kanuti NWR(D), Koyukuk NWR (E), North Slope (F), Selawik NWR (G), Point Lay (H), Anderson River (I), Mackenzie River (J), Kent Peninsula (K), Victoria Island (L), Queen Maud Gulf (M), Rasmussen Basin (N), Old Crow Flats, Yukon (O), Greenland (P), Anadyr Lowlands (Q), Kolyma River (R), Lena River (S), Magadan (T), Taimyr Peninsula (U), Vaygach Island (V), and Yana River (W).

When testing the different subspecies classifications (excluding Greenland and tule goose), the best grouping was proposed by Delacour (1954) with North America populations (currently classified as *A. a. frontalis*) and eastern Palearctic (with break at Lena River) grouped together and the western Palearctic as a second group; although it was marginally significant (Φ_CT_ = 0.135, *p *=* *0.06, Table 3). If the break between Palearctic regions was placed at the Kolyma River Delta, 9.9% (*p *=* *0.027) of the genetic variation was explained. In general, while flyway, geographic or subspecies groupings typically accounted for approximately 8%–14% of the genetic variation, AMOVAs showed that genetic variation was to a large extent partitioned within populations/localities (>50%).

**Figure 5:**
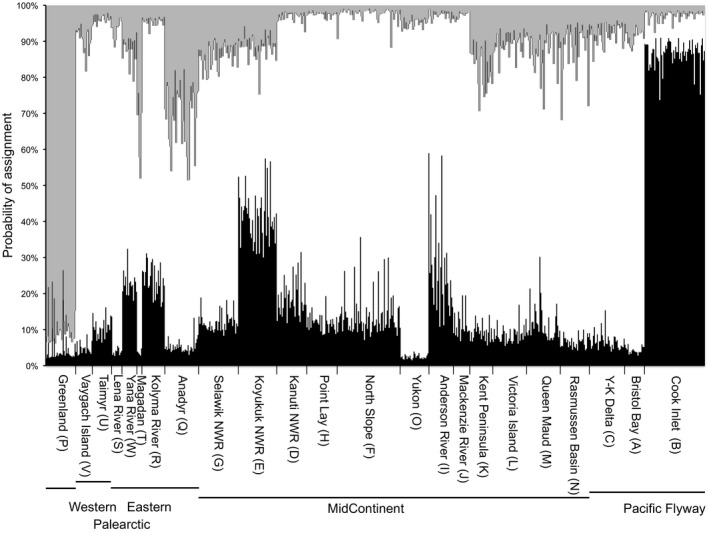
STRUCTURE analysis showing posterior probability assignment of individuals to each genetic cluster (*K *=* *3) using LOCIPRIOR (*r *<* *1). Letters in parentheses indicated population codes used in Figure 2

In contrast to mtDNA sequence data, we found very little structure within the microsatellite dataset. Significant pairwise *F*_ST_ estimates were restricted to comparisons with Greenland and Cook Inlet populations (Table 2). This general lack of genetic differentiation was also reflected in the STRUCTURE analysis where the most likely number of genetic clusters was *K *=* *3 deemed by both Evanno's method (Δ*K* = 5.0) and assessing Ln *P*(*K*) (*K *=* *3; −13,317.7 vs. *K *=* *1; −13414.6) using the LOCIPRIOR (*r *<* *1). When no location information prior was used, the most likely number of genetic clusters was one. The three genetic clusters corresponded with *F*_ST_ estimates whereby Greenland and Cook Inlet individuals were grouped in separate clusters with the remaining populations from Asia and North America representing a single cluster or showing admixture (Figure 5). Further, AMOVA groupings revealed small but significant genetic partitioning (<1% of variance) explained by multiple groupings (e.g., flyway, geographic proximity, Old vs. New World, and subspecies; Table 3 and Supporting Information Appendix S4); while >95% of total genetic variation was partitioned within populations.

### 3.3 Demographic history and gene flow based on mtDNA

**Figure 6:**
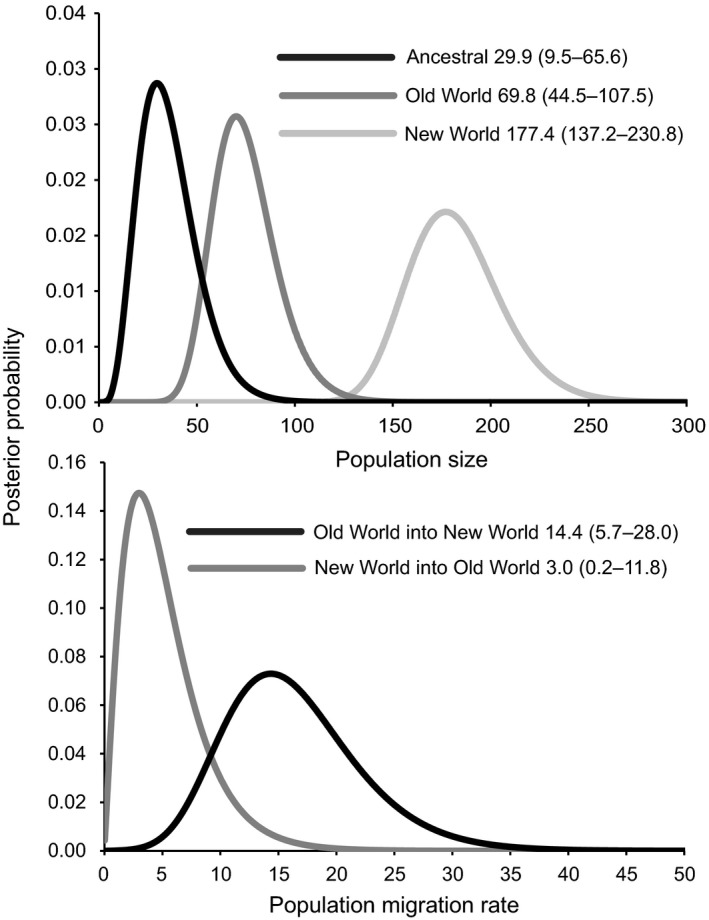
Estimates of the population size (Ɵ; top) and number of migrants per generation (population migration rate; bottom) for Old World (Eurasia) and New World (North America) greater white‐fronted goose populations with lower and upper bound of the estimated 95% higher posterior interval (HPD) indicated. Please see Figure 2 and Table 1 for populations included in each of these two groups. In general, samples included in the Old World include population codes Q–W and New World includes population codes A–N

In general, the population size parameter (ϴ) was larger than the ancestral effective population size (Figure 6). The population size parameter for New World populations was approximately five times larger than Old World (not including Greenland), suggesting a clear population expansion in North America. Although the population size was twice as large in the Old World relative to ancestral population size, suggestive of population expansion, the HPD95 ranges overlapped, potentially indicating population stasis after divergence. This lack of clear demographic expansion was also evident in the neutrality tests, Fu's *F*_s_ and Tajima's *D*. Significant negative test statistics, indicating population expansion, were observed in Kolyma, Russia and Selawik, Alaska (Fu's *F*_s_) and Yana River Delta in Russia (Tajima's *D*).

Prominent traces of gene flow were detected from New World populations into Old World populations (2*Nm* = three migrants per generation) and in the reverse direction (2*Nm* = 14 migrants per generation). Although the peak values showed a strong asymmetrical direction of gene flow from Old World into New World, confidence intervals broadly overlapped (Figure 6). The time of divergence parameter (*t*) peaked at 1.518, which when converted to years (assuming a generation time of 6 years) suggests that Old and New World lineages diverged around 86,000 years before the present (50,000–144,000 ybp), prior to the peak of the last glacial maximum.

### 3.4 Band recovery data

Band recovery data showed that greater white‐fronted geese had a high degree of fidelity to the flyway and the continent where they were initially captured (Supporting Information Appendix S2). We observed only four instances of intercontinental dispersal and less than 1% of flyway switching within North America or Eurasian flyways.

## 4 DISCUSSION

We found significant differentiation based on mtDNA control region sequence data, and more limited differentiation based on microsatellite fragment data, across all greater white‐fronted goose sampling locations, with populations nesting on Greenland and in Cook Inlet (tule goose) being the most distinctive. The high mtDNA structure, and general lack of nuclear DNA structure is a common finding in waterfowl; however, this is one of the first studies in waterfowl to our knowledge to show that proposed flyway delineation corresponds to a significant partitioning in mtDNA genetic variance (Kraus et al., 2014; Liu et al., 2012; Peters & Omland, 2007). Unlike for brant, the other circumpolar goose species, and some other Arctic bird species (see Henningson & Alerstam, 2008), migration routes used by greater white‐fronted geese are maintained within the same continent, which is reflected by the lack of intercontinental band recoveries and low mtDNA haplotype sharing across continents. Within waterfowl, geese and swans are unique in their long‐lasting parental associations, and some greater white‐fronted geese have familial associations lasting for 8 years or more (Ely, 1993; Warren, Fox, Walsh,  & O'Sullivan, 1993). Thus cultural transmission of migratory tendencies likely plays a role in the maintenance and integrity of traditional migratory routes as well as the distribution of genetic variation.

### 4.1 Maintenance of flyway structure

Our analysis based on mtDNA showed significant structure which can at least be partially be explained by flyway designation (Table 3). The general lack of haplotype sharing across major flyway designations in North America (6 out of 120 haplotypes) and Palearctic flyways (four of 51 haplotypes) suggests restricted effective female dispersal (gene flow) and strong migratory connectivity on the broad‐scale. This coincides with banding data which shows less than 1% of banded geese switched flyways, at least temporally as observed in the four Greenland greater white‐fronted goose intercontinental records (Supporting Information Appendix S2) and within pink‐footed goose (*A. brachyrhynchos*; Madsen et al., 2014). Although banding records only represent a small proportion of the population and therefore may underestimate the true level of flyway abmigration, there are copious recoveries of many other species of North American waterfowl in eastern Eurasia, especially brant, lesser snow geese, *Chen caerulescens*, and northern pintail, *Anas acuta* (Flint et al., 2009; Henny, 1973). This suggests that broad‐scale dispersal may be relatively lower in greater white‐fronted geese compared to other species which may account for the low mtDNA haplotype sharing. Further, most shared haplotypes were more interior or central in the network, suggesting they may be retained ancestral haplotypes rather the result of current ongoing gene flow.

Assuming a low level of flyway switching within and across continents results in gene flow, this small percentage of abmigration could be enough to maintain genetic connectivity evident by the long‐term gene flow between Old (Eurasia) and New World (North America). The pattern (lower structure in microsatellite loci relative to mtDNA) could be due to a greater level of philopatry in females, with gene flow largely mediated by males as suggested for the near panmictic population structure across the Arctic at nuclear markers for some dabbling duck species (see Kraus et al., 2014; Peters et al., 2014). Here we only observed significant nuclear differentiation based on *F*_ST_ involving the Cook Inlet and Greenland populations while observing panmixia across the rest of the species’ range. It should be noted that these two populations have very restricted breeding areas and low population census sizes (<10,000 Cook Inlet and 18,800 for Greenland) compared to other regions (2.6 million for North American midcontinent and 685,000 for Pacific Flyway; CAFF, 2018). Incomplete lineage sorting, therefore, may also play a role in the observed pattern, and hypothesis is supported by the ubiquity and high frequency of the most common allele(s) for each microsatellite locus across regions. Given more recent coalescent times associated with microsatellite loci and the potential connectivity via male dispersal, we suggest that a SNP‐based approach may be needed to achieve the necessary statistical power to investigate within flyway structure (Jonker et al., 2012); however, is likely a combination of both gene flow (e.g., within flyway) and incomplete lineage sorting (e.g., across continents) is influencing the pattern of genetic diversity of greater white‐fronted geese.

Greater white‐fronted geese possess many behavioral and ecological characteristics that might restrict genetic interchange among populations, not only at the broad flyway (macrogeographic) scale but also on a more local (microgeographic) scale (see Ely et al., 2017). As seen in Pacific Flyway white‐fronted geese and other goose species, the timing of pairing can have pronounced implications on genetic structuring in geese (Ely & Scribner, 1994; Ely et al., 2017). Typically, in waterfowl, multiple populations from disparate locales utilize the same wintering site, thus providing an avenue for genetic exchange if pair formation occurs during this period of the annual cycle. While ducks form pair bands in winter, pair formation in some geese, including greater white‐fronted geese, likely occurs during the spring and summer; this limits the choice of mates to birds at local staging and breeding areas or shared molting sites. Further, North American greater white‐fronted goose populations are temporally segregated throughout much of the annual cycle despite having a high degree of spatial overlap during winter (Ely & Takekawa, 1996; Ely et al., 2013). This temporal and spatial segregation is more pronounced in the Pacific Flyway with segregation of site use observed at the population level (Ely et al., 2017), while the midcontinent population tends to be more temporally segregated by general breeding areas (Ely et al., 2013). In the midcontinent, Ely et al. (2013) found that taiga‐nesting birds (including interior Alaska and Old Crow Flats, Canada) tended to migrate earlier than tundra‐nesting populations, while eastern Arctic populations generally wintered farther east (Louisiana) than other midcontinent populations. This difference in degree of segregation between flyways is reflected in our pairwise *F*_ST_ comparisons, as all Pacific Flyway populations showed significant mtDNA differentiation (also see Ely et al., 2017) while there was a general lack of differentiation across Alaska and western Canada tundra‐nesting populations.

### 4.2 Demographic history

The greater white‐fronted goose is the only representative of the “gray” geese in North America and is thought to have a Eurasian origin (Ottenburghs et al., 2016). Our isolation‐with‐migration analysis suggests that North American and Eurasian populations began diverging at least 50,000 years ago, with post‐divergence gene flow biased toward movement into North America. Directionality in gene flow and pronounced population expansion in North America further suggests an Old World origin for this species complex as observed in many other species where Beringia was used as an entrance into North America (e.g., Waltari, Hoberg, Lessa, & Cook, 2007). In North America, at the start of the interglacial period approximately 14,000 years ago, an ice‐free corridor was established between the Cordilleran and Laurentide ice sheets (Adams, 1997; Pielou, 1991). As proposed in red knots (*Calidris canutus*; Buehler, Baker, & Piersma, 2006), this ice‐free opening may have been used to develop new migration routes to southern North America which today would equate to the flyways still used by the midcontinent (Mississippi and Central Flyway) and Pacific Flyway greater white‐fronted geese populations; although this remains to be tested.

Ploeger (1968) proposed four potential refugia for greater white‐fronted goose during the last glacial period: (a) western Europe, (b) Russia and western Siberia, (c) eastern Siberia and Bering Sea, and (d) northwest Arctic Canadian Archipelago. These proposed refugia roughly correspond to areas of transitioning morphology in the greater white‐fronted goose; however, Ploeger hypothesized the Canadian Archipelago as a possible refugium for the tule goose before it was known to breed only in south central Alaska. Ploeger further hypothesized that Beringian populations during the last post glacial period could have expanded both eastward and westward, which is supported by our finding of the highest genetic diversity in central Beringia. This also suggests a potential common origin for both eastern Palearctic and North American populations.

The Lena River is generally accepted to be the westernmost boundary of Beringia and is a well‐known suture zone (Hewitt, 2004). MtDNA genetic diversity of greater white‐fronted geese within Eurasia is at its lowest in the vicinity of the suture zone with western Palearctic sampled populations (Taimyr Peninsula to Vaygach Island) potentially representing a second genetic cline. This suggests multiple refugia in Eurasia, where the ice caps of Taimyr Peninsula and Putarana Mountains separated these two refugial areas during the Weichselican Glaciation (Ávila‐Jiménez & Coulson, 2011; Möller, Alexanderson, Funder, & Hjort, 2015; Ploeger, 1968) with possible secondary contact at Lena River and Yana River as populations began to expand and intermix, as suggested by the high concentration of individuals sharing haplotypes between Palearctic flyways uncovered this area. This may explain the nonsignificant mtDNA differentiation between Lena River and Yana River populations with their nearest sampling localities to the east and west.

The same genetic pattern is seen in North America at the Mackenzie River (the easternmost boundary of Beringia; Hultén, 1937) and Anderson River populations. These two locales also lie near a known suture zone (Hewitt, 2004) in which greater white‐fronted geese share haplotypes with Arctic Alaska and Canada, and there is general lack of genetic differentiation within the central part of their Nearctic distribution (Kent Peninsula to North Slope Alaska). These data are consistent with Ploeger's hypothesis that geese occupying greater Beringia during the last glacial maximum expanded both eastward and westward, eventually coming into secondary contact with populations expanding out of different refugia in eastern Nearctic locales, and western European locales, respectively.

### 4.3 Taxonomic implications

The subspecies concept and its usefulness has been a subject of controversy in ornithology for decades (Mayr, 1942; Winker, 2010; see review in Ornithological Monographs No. 67). The taxonomy of the greater white‐fronted goose is a prime example of this, as its taxonomic history has been highly debated and confusing, with respect to the number of subspecies and their nomenclature (see Banks, 2011). As subspecies rank can often be used to inform and be the basis of conservation policies (Winker, 2010), understanding the relationships between the distribution of genetic diversity and subspecies designations can be crucial. As mentioned by Zink and Dittman (1992) and reiterated by Cicero and Johnson (2006), analyses of geographic variation and potential subsequent delineation into subspecies must be done with known breeding origins. Much of the confusion surrounding the subspecific status of greater white‐fronted geese likely centers from the use of wintering specimens for taxonomic determinations (Ely & Dzubin, 1994) and mean morphological differences in subspecies descriptions, where more rigorous testing is needed (Cicero & Johnson, 2006; Patten & Unitt, 2002).

The classification of Cook Inlet (tule; *A. a. elagsi*) and Greenland (*A. a. flavirostris*) populations as valid subspecies has been little challenged, given their large body size and darker coloration, with slight overlap with other populations (Ely et al., 2005), and especially their isolated breeding areas. These multiple lines of evidence suggest these two populations are on independent evolutionary trajectories, and therefore, it is not surprising that only geese from these two locales showed significant genetic differentiation at both marker types. Both subspecies have restricted ranges throughout their life cycle and reduced population sizes, relative to the more broadly distributed proposed subspecies with which they overlap in distribution to some degree during winter. As such, the genetic data support morphological, ecological and behavioral evidence that provided the foundation of the subspecies hypotheses associated with these two discrete breeding populations. We note that the tule goose is considered to be at risk by the International Waterfowl Research Bureau (Alaska Department of Fish and Game, 2015; Green, 1996), and the Greenland greater white‐fronted goose is one of the first subspecies to be red listed in the United Kingdom by the Birds of Conservation Concern assessment (Eaton et al., 2009).

Nevertheless, while the tule goose and Greenland white‐fronted goose exhibit distinct phenotypes, there is considerable morphological variation between and within the proposed *A. a. frontalis* and *A. a. albifrons* subspecies, obfuscating subspecies determinations (Ely et al., 2005). For example, eastern Palearctic populations have been designated as three different subspecies (*A. a. albifrons* Hartlaub, 1852; *A. a. albicans* Mooij & Zöckler, 2000; and *A. a. frontalis* Delacour, 1954) based on morphology or migratory behavior. Our genetic results do not resolve this issue. AMOVA analyses suggest eastern Palearctic birds (Figure 2; letters Q–T, W) show a close affiliation to North American midcontinent populations (Figure 2; letters D–O), to which they show morphologically similarities (Delacour, 1954). However, the amount of mtDNA genetic variation explained when eastern Palearctic are grouped by themselves (flyway AMOVA grouping) explained approximately the same amount of mtDNA genetic variation (11% vs. 13%) as that under the subspecies model (subspecies AMOVA grouping, see Table 3). In addition, we found no genetic support for the proposed subspecies (*A. a. sponsa*; Banks, 2011; but see Orthmeyer et al., 1995), based on their smaller average body size, encompassing the Yukon‐Kuskokwim Delta and Bristol Bay regions. Due to the lack of clear distinction in subspecies attributions, based on mtDNA sequence data and the presence of multiple body sizes along with a clear phenotypic body size cline in Eurasia, a genomic approach may be needed to determine if the different phenotypes represent genetically discrete populations, and to test taxonomic designations used to make management prescriptions.

## CONFLICT OF INTEREST

None declared.

## AUTHOR CONTRIBUTIONS

REW, CRE, and SLT were involved in study design. REW collected and analyzed genetic data, and CRE collected data and analyzed banding data. REW lead the writing. All authors contributed to writing and interpretation of results. All authors approve the final version of the manuscript.

## DATA ACCESSIBILITY

Microsatellite genotype data and sample information are available in Wilson (2018; https://doi.org/10.5066/f71g0jgn). Mitochondrial control region sequences are available on GenBank (Accession Numbers: KY704180–KY704263, MH306217–MH306650).
