# Supplementary material for: Flyway structure in the circumpolar greater white‐fronted goose
Source: Ecol Evol. 2018 Jul 30;8(16):8490–507. doi: 10.1002/ece3.4345 (PMC6144976; doi:10.1002/ece3.4345)
Supplement: Supplementary file 1 [file ECE3-8-8490-s001.docx]

Appendix S1. Phylogenetic reconstruction of greater white-fronted goose populations based on mtDNA control-region estimated using Bayesian approach in BEAST (Heled & Drummond 2010). Numbers at nodes are Bayesian posterior probabilities. Phylogeny was edited using the program MrEnt (Zuccon & Zucconm 2013). Population codes used in Figure 1 and Table 1 are given after each population name in parentheses.

Eurasia

North America

Greenland

North America

References

Heled, J., & Drummond, A. J. (2010). Bayesian inference of species trees from multilocus data. *Molecular Biology and Evolution*, **27**, 570–580.

Zuccon A., & Zucconm D. (2013). MrEnt v.2.5. Program distributed by the authors. [http://www.mrent.org].
