# Supplementary material for: Flyway structure in the circumpolar greater white‐fronted goose
Source: Ecol Evol. 2018 Jul 30;8(16):8490–507. doi: 10.1002/ece3.4345 (PMC6144976; doi:10.1002/ece3.4345)
Supplement: Supplementary file 2 [file ECE3-8-8490-s002.docx]

Appendix S2. Compilation of band recovery data and estimates of flyway switching and inter-continental exchange of greater white-fronted geese in North America and Eurasia.

**Methods**

We obtained banding and recovery data from the U. S. Geological Survey (USGS) Bird Banding Laboratory (BBL) in Laurel, Maryland, for greater white-fronted geese, which included data from birds banded or recovered in North America through 2015. Publicly accessible BBL records generally do not include information on birds banded outside of the United States and Canada. However, we contacted the BBL directly (D. Bystrak, pers comm. 29 Dec 2015) concerning any interchange of foreign-banded greater white-fronted geese recovered in North America, or North American-banded greater white-fronted geese that may have been encountered outside North America. To determine flyway and continental fidelity of greater white-fronted geese in Eurasia we contacted the Russian Ringing Centre (K. Litvin, pers. com.), and accessed Euring data (T. Fox, pers. com.). Banding records came from geese captured on breeding and wintering areas, but there were very few recoveries (or observations) on northern breeding areas. We relied on published geographic distribution accounts for white-fronted geese to assess emigration events (BirdLife International, 2016).

**Results**

No recoveries of geese initially banded in North America (n=25,871) have been from outside North America, nor have there been any recoveries of geese initially banded in Eurasia (n=5,530) outside of Eurasia. Four of the 704 (0.57%) recoveries of Greenland-banded greater white-fronted geese have been in North America. Flyway fidelity has also been high, with only one of 5,518 (< 0.01%) recoveries of geese marked in the western Palearctic being recovered in the eastern Palearctic, and no recoveries of eastern Palearctic banded birds in the western Palearctic. Fidelity across flyways in North America was also quite high, with 26 of 15,485 (0.17%) recoveries of geese initially marked in the Central or Mississippi flyways being recovered in the Pacific Flyway and only 7 of 1,128 (0.62%) recoveries of geese initially marked in the Pacific Flyway being recovered in the other North American Flyways. There was however a large degree of overlap across flyways among geese initially banded at a Midcontinent mixed molting site in interior Alaska, where 376 of 2,375 (15.83%) of the recoveries were in the Pacific Flyway. This is the only northern site we are aware of that was used by greater white-fronted geese from >1 flyway.

Table S2-1. Intercontinental and inter-flyway movement of greater white-fronted geese as determined from recovery distribution of leg-banded birds.

|  |  | Location of Recovery (continent or flyway) | | |
| --- | --- | --- | --- | --- |
| Banding Location | Number of recoveries | Intra | Inter | % interchange |
|  | |  |  |  |
| *INTER-CONTINENTAL*^a^ | |  |  |  |
| **North America x Greenland** | |  |  |  |
| North America | 25,871 | 25,871 | 0 | 0.00 |
| Greenland/Ireland^b^ | 704 | 700 | 4 | 0.57 |
|  | |  |  |  |
| **North America x Eurasia** | |  |  |  |
| *North America*^c^ | 25,871 | 25,871 | 0 | 0.00 |
| *Eurasia*^d^ | 5,530 | 5,530 | 0 | 0.00 |
|  |  |  |  |  |
| *INTER-FLYWAY* |  |  |  |  |
| **North American flyways**^e^ | |  |  |  |
| *Midcontinent*^f^ | 15,485 | 15,459 | 26 | 0.17 |
| *Pacific*^g^ | 1,128 | 1,121 | 7 | 0.62 |
| Mixed molting site^h^ | 2,375 | 1,998^h^ | 376 | 15.83 |
|  |  |  |  |  |
| **Eurasian flyways** |  |  |  |  |
| *Western Palearctic*^i^ | 5,518 | 5,517 | 1 | 0.00 |
| *Eastern Palearctic*^j^ | 27 | 27 | 0 | 0.00 |

^a^ Includes all recoveries (shot or found dead) of geese banded on breeding, staging and wintering areas.

^b^ Data from Greenland, Iceland, UK, and Ireland only includes Greenland (*A. a. flavirostris*) birds (A. Fox, pers. comm.)

^c^ Confirmation of no foreign recoveries of North American origin from Danny Bystrak (USGS Bird Banding Laboratory December 2015).

^d^ Data from EURING, including Belgium, Denmark, Germany (3 different schemes), Hungary, Netherlands, Poland, Sweden, Switzerland and United Kingdom (excluding Greenland *flavirostris* race birds).

^e^ Recoveries for inter-flyway comparison in North America restricted to birds banded on northern breeding and molting areas and shot or found dead. Recoveries of geese in central Mexico not included in analysis given overlap between flyways in that area. Does not include 9 recoveries of banded birds in the Atlantic Flyway (7 originally banded at midcontinent banding sites and 2 on the mixed molting site between midcontinent and Pacific).

^f^ Includes tundra and taiga breeding birds banded at northern breeding and molting areas and affiliated with the Central and Mississippi Flyways (Ely et al. 2013).

^g^ Includes birds banded on the Yukon-Kuskokwim Delta, Bristol Bay, and Cook Inlet, Alaska.

^h^ Known molting area of birds from Interior Alaska and Pacific Flyway (no locals banded). Includes Innoko River Valley and wetlands along the Yukon River near Holy Cross, AK. Intra-flyway recovery for this specific instance refers to birds recovered in midcontinent areas and “Inter” to birds recovered in the region of Pacific flyway.

^i^ Western Palearctic includes Russia (west of the Khatanga River), The Netherlands, Germany, UK, France, Lithuania, Bulgaria, Hungary, and Sweden. (Russian bandings and recoveries from Bird Ringing Centre of Russia, IEE RAS – February 2016; K. Litvin).

^j^ Eastern Palearctic includes Russia (east of the Khatanga River), Japan, China, and Korea*.* Russian bandings and recoveries from Bird Ringing Centre of Russia, IEE RAS – February 2016; K. Litvin.

**References**

BirdLife International. (2016). *Anser albifrons*. The IUCN red list of threatened species 2016: e.T22679881A85980652. http://dx.doi.org/10.2305/IUCN.UK.2016-3.RLTS.T22679881A85980652.en. Downloaded on 04 December 2017.

Ely, C.R., Neiman, D.J., Alisauskas, R.T., Schmutz, J.A., & Hines, J.E. (2013). Geographic variation in migration chronology and winter distribution of midcontinent Greater white-fronted geese. *Journal of Wildlife Management*, **77**, 1182-1191.
