# Supplementary material for: Flyway structure in the circumpolar greater white‐fronted goose
Source: Ecol Evol. 2018 Jul 30;8(16):8490–507. doi: 10.1002/ece3.4345 (PMC6144976; doi:10.1002/ece3.4345)
Supplement: Supplementary file 4 [file ECE3-8-8490-s004.docx]

Appendix S4. Hierarchical analysis of molecular variance of allelic and haplotypic frequencies to test hypotheses associated with 1) subspecies classification schemes; 2) flyway designation; 3) geographic proximity; 4) nesting habitat; 5) putative refugia for greater white-fronted goose populations. Some populations were not included in all groupings as it would be the sole representative for that group. For example, Cook Inlet and Greenland populations were excluded from subspecies grouping analysis as they are the only members of their respective subspecies. Significant fixation indices (P < 0.05) are indicated in bold. Please refer to Figure 1 and Table 1 for location of each population^1^.

|  |  | **Variance Components** | | | | |
| --- | --- | --- | --- | --- | --- | --- |
| **Model** | **Hypothesized Groupings** | **F_ST_** | **F_SC_** | **F_CT_** | **%**  **among groups** | ***P*_(among group)_** |
|  | **Microsatellite Loci** |  |  |  |  |  |
| Subspecies  (Hartlaub 1852) | [Bristol Bay, Y-K Delta, Point Lay, North Slope, Anderson, Mackenzie, Koyukuk, Kanuti, Selawik, Yukon, Kent, Queen Maud, Victoria, Rasmussen] [Kolyma, Anadyr, Magadan, Yana, Lena, Taimyr, Vaygach] | **0.010** | **0.006** | **0.004** | 0.40 | < 0.05 |
| Subspecies  (Delacour 1954) | [Bristol Bay, Y-K Delta, Point Lay, North Slope, Anderson, Mackenzie, Koyukuk, Kanuti, Selawik, Yukon, Kent, Queen Maud, Victoria, Rasmussen, Kolyma, Anadyr, Magadan, Yana, Lena] [Taimyr, Vaygach] | **0.012** | **0.007** | 0.006 | 0.60 | 0.052 |
| Subspecies | [Bristol Bay, Y-K Delta, Point Lay, North Slope, Anderson, Mackenzie, Koyukuk, Kanuti, Selawik, Yukon, Kent, Queen Maud, Victoria, Rasmussen, Kolyma, Anadyr, Magadan, Yana] [Taimyr, Vaygach, Lena] | **0.006** | **0.013** | **0.007** | 0.70 | 0.013 |
| Subspecies | [Bristol Bay, Y-K Delta, Point Lay, North Slope, Anderson, Mackenzie, Koyukuk, Kanuti, Selawik, Yukon, Kent, Queen Maud, Victoria, Rasmussen, Kolyma, Anadyr, Magadan] [Taimyr, Vaygach, Yana, Lena] | **0.010** | **0.007** | **0.003** | 0.30 | < 0.05 |
| Subspecies  (Mooij and Zockler 2000) | [Bristol Bay, Y-K Delta, Point Lay, North Slope, Anderson, Mackenzie, Koyukuk, Kanuti, Selawik, Yukon, Kent, Queen Maud, Victoria, Rasmussen] [Kolyma, Anadyr, Magadan, Yana, Lena] [Taimyr, Vaygach] | **0.010** | **0.005** | **0.005** | 0.50 | < 0.05 |
| Subspecies  (Banks 2011) | [Bristol Bay, Y-K Delta] [Point Lay, North Slope, Anderson, Mackenzie, Koyukuk, Kanuti, Selawik, Yukon, Kent, Queen Maud, Victoria, Rasmussen] [Taimyr, Vaygach, Kolyma, Anadyr, Magadan, Yana, Lena] | **0.009** | **0.006** | **0.003** | 0.30 | < 0.05 |
| Old vs New World | [Bristol Bay, Y-K Delta, Cook Inlet, Point Lay, North Slope, Anderson, Mackenzie, Koyukuk, Kanuti, Selawik, Yukon, Kent, Queen Maud, Victoria, Rasmussen] [Taimyr, Vaygach, Kolyma, Anadyr, Magadan, Yana, Lena] | **0.015** | **0.010** | **0.005** | 0.50 | < 0.05 |
| Old vs New World | [Bristol Bay, Y-K Delta, Cook Inlet, Point Lay, North Slope, Anderson, Mackenzie, Koyukuk, Kanuti, Selawik, Yukon, Kent, Queen Maud, Victoria, Rasmussen] [Greenland, Taimyr, Vaygach, Kolyma, Anadyr, Magadan, Yana, Lena] | **0.019** | **0.015** | **0.005** | 0.50 | < 0.05 |
| Old vs New World | [Bristol Bay, Y-K Delta, Cook Inlet, Point Lay, North Slope, Anderson, Mackenzie, Koyukuk, Kanuti, Selawik, Yukon, Kent, Queen Maud, Victoria, Rasmussen, Greenland] [Taimyr, Vaygach, Kolyma, Anadyr, Magadan, Yana, Lena] | **0.019** | **0.015** | **0.004** | 0.40 | < 0.05 |
| Flyway | [Vaygach, Taimyr] [Lena, Yana, Kolyma, Magadan, Anadyr] [Point Lay, North Slope, Anderson, Mackenzie, Koyukuk, Kanuti, Selawik, Yukon, Kent, Queen Maud, Victoria, Rasmussen] [Y-K Delta, Bristol Bay, Cook Inlet] | **0.014** | **0.009** | **0.006** | 0.60 | 0.009 |
| Flyway | [Vaygach, Taimyr, Lena, Yana] [Kolyma, Magadan, Anadyr] [Point Lay, North Slope, Anderson, Mackenzie, Koyukuk, Kanuti, Selawik, Yukon, Kent, Queen Maud, Victoria, Rasmussen] [Y-K Delta, Bristol Bay, Cook Inlet] | **0.014** | **0.009** | **0.005** | 0.50 | 0.006 |
| Flyway | [Vaygach, Taimyr] [Kolyma, Magadan, Anadyr] [Point Lay, North Slope, Koyukuk, Kanuti, Selawik, Yukon, Anderson, Mackenzie, Kent, Queen Maud, Victoria, Rasmussen] [Y-K Delta, Bristol Bay, Cook Inlet] | **0.014** | **0.009** | **0.006** | 0.60 | 0.018 |
| Geographic | [Greenland, Vaygach, Taimyr, Lena] [Yana, Kolyma, Magadan, Anadyr] [Point Lay, North Slope, Anderson, Mackenzie, Koyukuk, Kanuti, Selawik, Yukon, Kent, Queen Maud, Victoria, Rasmussen] [Y-K Delta, Bristol Bay, Cook Inlet] | **0.018** | **0.013** | **0.005** | 0.50 | < 0.05 |
| Geographic | [Vaygach, Taimyr, Lena] [Yana, Kolyma, Magadan, Anadyr] [Point Lay, North Slope, Anderson, Mackenzie, Koyukuk, Kanuti, Selawik, Yukon, Kent, Queen Maud, Victoria, Rasmussen, Greenland] [Y-K Delta, Bristol Bay, Cook Inlet] | **0.018** | **0.014** | 0.004 | 0.40 | 0.053 |
| Geographic | [Greenland, Vaygach, Taimyr, Lena, Yana] [Kolyma, Magadan, Anadyr] [Point Lay, North Slope, Anderson, Mackenzie, Koyukuk, Kanuti, Selawik, Yukon, Kent, Queen Maud, Victoria, Rasmussen] [Y-K Delta, Bristol Bay, Cook Inlet] | **0.018** | **0.013** | **0.005** | 0.50 | 0.023 |
| Geographic | [Vaygach, Taimyr, Lena, Yana] [Kolyma, Magadan, Anadyr] [Point Lay, North Slope, Anderson, Mackenzie, Koyukuk, Kanuti, Selawik, Yukon, Kent, Queen Maud, Victoria, Rasmussen, Greenland] [Y-K Delta, Bristol Bay, Cook Inlet] | **0.018** | **0.014** | 0.004 | 0.40 | 0.077 |
| Geographic | [Vaygach, Taimyr] [Lena, Yana] [Kolyma, Magadan, Anadyr] [Point Lay, North Slope, Koyukuk, Kanuti, Selawik, Yukon, Anderson, Mackenzie, Kent, Queen Maud, Victoria, Rasmussen, Greenland] [Y-K Delta, Bristol Bay, Cook Inlet] | **0.017** | **0.017** | 0.001 | 0.10 | 0.365 |
| Geographic | [Greenland, Vaygach, Taimyr, Lena, Yana, Kolyma, Magadan, Anadyr] [Point Lay, North Slope, Koyukuk, Kanuti, Selawik, Yukon, Anderson, Mackenzie] [Kent, Queen Maud, Victoria, Rasmussen] [Y-K Delta, Bristol Bay, Cook Inlet] | **0.017** | **0.013** | **0.004** | 0.40 | 0.014 |
| Geographic | [Vaygach, Taimyr, Lena, Yana, Kolyma, Magadan, Anadyr] [Point Lay, North Slope, Koyukuk, Kanuti, Selawik, Yukon, Anderson, Mackenzie] [Kent, Queen Maud, Victoria, Rasmussen, Greenland] [Y-K Delta, Bristol Bay, Cook Inlet] | **0.018** | **0.013** | **0.005** | 0.50 | 0.004 |
| Geographic | [Greenland, Vaygach, Taimyr, Lena, Yana] [Kolyma, Magadan, Anadyr] [Point Lay, North Slope, Koyukuk, Kanuti, Selawik, Yukon, Anderson, Mackenzie] [Kent, Queen Maud, Victoria, Rasmussen] [Y-K Delta, Bristol Bay, Cook Inlet] | **0.017** | **0.013** | **0.004** | 0.40 | 0.024 |
| Geographic | [Vaygach, Taimyr, Lena, Yana] [Kolyma, Magadan, Anadyr] [Point Lay, North Slope, Koyukuk, Kanuti, Selawik, Yukon, Anderson, Mackenzie] [Kent, Queen Maud, Victoria, Rasmussen, Greenland] [Y-K Delta, Bristol Bay, Cook Inlet] | **0.017** | **0.013** | **0.004** | 0.40 | 0.015 |
| Geographic | [Greenland, Vaygach, Taimyr] [Lena, Yana, Kolyma, Magadan, Anadyr] [Point Lay, North Slope, Anderson, Mackenzie] [Koyukuk, Kanuti, Selawik, Yukon] [Kent, Queen Maud, Victoria, Rasmussen] [Y-K Delta, Bristol Bay, Cook Inlet] | **0.017** | **0.013** | **0.005** | 0.50 | 0.006 |
| Geographic | [Vaygach, Taimyr] [Lena, Yana, Kolyma, Magadan, Anadyr] [Point Lay, North Slope, Anderson, Mackenzie] [Koyukuk, Kanuti, Selawik, Yukon] [Kent, Queen Maud, Victoria, Rasmussen, Greenland] [Y-K Delta, Bristol Bay, Cook Inlet] | **0.018** | **0.013** | **0.005** | 0.50 | 0.024 |
| Geographic | [Greenland, Vaygach, Taimyr, Lena, Yana] [Kolyma, Magadan, Anadyr] [Point Lay, North Slope, Anderson, Mackenzie] [Koyukuk, Kanuti, Selawik, Yukon] [Kent, Queen Maud, Victoria, Rasmussen] [Y-K Delta, Bristol Bay, Cook Inlet] | **0.017** | **0.014** | 0.003 | 0.30 | 0.052 |
| Geographic | [Vaygach, Taimyr, Lena, Yana] [Kolyma, Magadan, Anadyr] [Point Lay, North Slope, Anderson, Mackenzie] [Koyukuk, Kanuti, Selawik, Yukon] [Kent, Queen Maud, Victoria, Rasmussen, Greenland] [Y-K Delta, Bristol Bay, Cook Inlet] | **0.017** | **0.014** | **0.003** | 0.30 | 0.027 |
| Nesting Habitat | [Greenland, Vaygach, Taimyr, Lena, Yana, Kolyma, Anadyr, Point Lay, North Slope, Bristol Bay, Cook Inlet, Y-K Delta, Anderson, Mackenzie, Kent, Queen Maud, Victoria, Rasmussen][Magadan, Cook Inlet, Koyukuk, Selawik, Kanuti, Yukon] | **0.018** | **0.015** | 0.003 | 0.30 | > 0.05 |
| Refugia (Ploeger 1968) | [Vaygach, Taimyr [Lena, Yana, Kolyma, Magadan, Anadyr, Point Lay, North Slope, Y-K Delta, Bristol Bay, Cook Inlet, Koyukuk, Kanuti, Selawik] [Anderson, Mackenzie, Yukon, Kent, Queen Maud, Victoria, Rasmussen] | **0.011** | **0.010** | 0.001 | 0.10 | > 0.05 |
| Refugia  (Ploeger 1968) | [Vaygach, Taimyr, Lena, Yana] [Kolyma, Magadan, Anadyr, Point Lay, North Slope, Y-K Delta, Bristol Bay, Cook Inlet, Koyukuk, Kanuti, Selawik] [Anderson, Mackenzie, Yukon, Kent, Queen Maud, Victoria, Rasmussen] | **0.011** | **0.010** | 0.001 | 0.10 | > 0.05 |
| Refugia  (Ploeger 1968) | [Vaygach, Taimyr, Lena, Yana] [Kolyma, Magadan, Anadyr, Point Lay, North Slope, Y-K Delta, Bristol Bay, Cook Inlet, Koyukuk, Kanuti, Selawik, Anderson, Mackenzie, Yukon] [Kent, Queen Maud, Victoria, Rasmussen] | **0.011** | **0.010** | 0.002 | 0.20 | > 0.05 |
|  |  |  |  |  |  |  |
|  | **MtDNA Control Region** | **Φ_ST_** | **Φ_SC_** | **Φ_CT_** | **%**  **among groups** | ***P*_(among group)_** |
| Subspecies  (Hartlaub 1852) | [Bristol Bay, Y-K Delta, Point Lay, North Slope, Anderson, Mackenzie, Koyukuk, Kanuti, Selawik, Yukon, Kent, Queen Maud, Victoria, Rasmussen] [Kolyma, Anadyr, Magadan, Yana, Lena, Taimyr, Vaygach] | **0.286** | **0.267** | 0.025 | 2.5 | 0.089 |
| Subspecies  (Delacour 1954) | [Bristol Bay, Y-K Delta, Point Lay, North Slope, Anderson, Mackenzie, Koyukuk, Kanuti, Selawik, Yukon, Kent, Queen Maud, Victoria, Rasmussen, Kolyma, Anadyr, Magadan, Yana, Lena] [Taimyr, Vaygach] | **0.357** | **0.257** | 0.135 | 13.5 | 0.059 |
| Subspecies | [Bristol Bay, Y-K Delta, Point Lay, North Slope, Anderson, Mackenzie, Koyukuk, Kanuti, Selawik, Yukon, Kent, Queen Maud, Victoria, Rasmussen, Kolyma, Anadyr, Magadan, Yana] [Taimyr, Vaygach, Lena] | **0.333** | **0.259** | **0.099** | 9.9 | 0.027 |
| Subspecies | [Bristol Bay, Y-K Delta, Point Lay, North Slope, Anderson, Mackenzie, Koyukuk, Kanuti, Selawik, Yukon, Kent, Queen Maud, Victoria, Rasmussen, Kolyma, Anadyr, Magadan] [Taimyr, Vaygach, Yana, Lena] | **0.320** | **0.259** | **0.082** | 8.2 | 0.025 |
| Subspecies  (Mooij and Zockler 2000) | [Bristol Bay, Y-K Delta, Point Lay, North Slope, Anderson, Mackenzie, Koyukuk, Kanuti, Selawik, Yukon, Kent, Queen Maud, Victoria, Rasmussen] [Kolyma, Anadyr, Magadan, Yana, Lena] [Taimyr, Vaygach] | **0.304** | **0.253** | **0.068** | 6.8 | 0.021 |
| Subspecies  (Banks 2011) | [Bristol Bay, Y-K Delta] [Point Lay, North Slope, Anderson, Mackenzie, Koyukuk, Kanuti, Selawik, Yukon, Kent, Queen Maud, Victoria, Rasmussen] [Taimyr, Vaygach, Kolyma, Anadyr, Magadan, Yana, Lena] | **0.293** | **0.254** | **0.052** | 5.2 | 0.031 |
| Old vs New World | [Bristol Bay, Y-K Delta, Cook Inlet, Point Lay, North Slope, Anderson, Mackenzie, Koyukuk, Kanuti, Selawik, Yukon, Kent, Queen Maud, Victoria, Rasmussen] [Taimyr, Vaygach, Kolyma, Anadyr, Magadan, Yana, Lena] | **0.305** | **0.292** | 0.018 | 1.8 | 0.138 |
| Old vs New World | [Bristol Bay, Y-K Delta, Cook Inlet, Point Lay, North Slope, Anderson, Mackenzie, Koyukuk, Kanuti, Selawik, Yukon, Kent, Queen Maud, Victoria, Rasmussen] [Greenland, Taimyr, Vaygach, Kolyma, Anadyr, Magadan, Yana, Lena] | **0.299** | **0.293** | 0.009 | 0.9 | 0.204 |
| Old vs New World | [Bristol Bay, Y-K Delta, Cook Inlet, Point Lay, North Slope, Anderson, Mackenzie, Koyukuk, Kanuti, Selawik, Yukon, Kent, Queen Maud, Victoria, Rasmussen, Greenland] [Taimyr, Vaygach, Kolyma, Anadyr, Magadan, Yana, Lena] | **0.304** | **0.291** | 0.019 | 1.9 | 0.113 |
| Flyway | [Vaygach, Taimyr] [Lena, Yana, Kolyma, Magadan, Anadyr] [Point Lay, North Slope, Anderson, Mackenzie, Koyukuk, Kanuti, Selawik, Yukon, Kent, Queen Maud, Victoria, Rasmussen] [Y-K Delta, Bristol Bay, Cook Inlet] | **0.322** | **0.256** | **0.093** | 9.3 | 0.005 |
| Flyway | [Vaygach, Taimyr, Lena, Yana] [Kolyma, Magadan, Anadyr] [Point Lay, North Slope, Anderson, Mackenzie, Koyukuk, Kanuti, Selawik, Yukon, Kent, Queen Maud, Victoria, Rasmussen] [Y-K Delta, Bristol Bay, Cook Inlet] | **0.327** | **0.246** | **0.108** | 10.8 | 0.003 |
| Flyway | [Vaygach, Taimyr] [Yana River, Lena River] [Kolyma, Magadan, Anadyr] [Point Lay, North Slope, Koyukuk, Kanuti, Selawik, Yukon, Anderson, Mackenzie, Kent, Queen Maud, Victoria, Rasmussen] [Y-K Delta, Bristol Bay, Cook Inlet] | **0.329** | **0.241** | **0.117** | 11.7 | 0.001 |
| Geographic | [Greenland, Vaygach, Taimyr, Lena] [Yana, Kolyma, Magadan, Anadyr] [Point Lay, North Slope, Anderson, Mackenzie, Koyukuk, Kanuti, Selawik, Yukon, Kent, Queen Maud, Victoria, Rasmussen] [Y-K Delta, Bristol Bay, Cook Inlet] | **0.316** | **0.258** | **0.078** | 7.8 | 0.007 |
| Geographic | [Vaygach, Taimyr, Lena] [Yana, Kolyma, Magadan, Anadyr] [Point Lay, North Slope, Anderson, Mackenzie, Koyukuk, Kanuti, Selawik, Yukon, Kent, Queen Maud, Victoria, Rasmussen, Greenland] [Y-K Delta, Bristol Bay, Cook Inlet] | **0.323** | **0.254** | **0.092** | 9.2 | 0.004 |
| Geographic | [ Greenland, Vaygach, Taimyr, Lena, Yana] [Kolyma, Magadan, Anadyr] [Point Lay, North Slope, Anderson, Mackenzie, Koyukuk, Kanuti, Selawik, Yukon, Kent, Queen Maud, Victoria, Rasmussen] [Y-K Delta, Bristol Bay, Cook Inlet] | **0.321** | **0.248** | **0.096** | 9.6 | 0.002 |
| Geographic | [Vaygach, Taimyr, Lena, Yana] [Kolyma, Magadan, Anadyr] [Point Lay, North Slope, Anderson, Mackenzie, Koyukuk, Kanuti, Selawik, Yukon, Kent, Queen Maud, Victoria, Rasmussen, Greenland] [Y-K Delta, Bristol Bay, Cook Inlet] | **0.328** | **0.247** | **0.108** | 10.8 | 0.001 |
| Geographic | [Vaygach, Taimyr] [Lena, Yana] [Kolyma, Magadan, Anadyr] [Point Lay, North Slope, Koyukuk, Kanuti, Selawik, Yukon, Anderson, Mackenzie, Kent, Queen Maud, Victoria, Rasmussen, Greenland] [Y-K Delta, Bristol Bay, Cook Inlet] | **0.331** | **0.240** | **0.119** | 11.9 | 0.001 |
| Geographic | [Greenland, Vaygach, Taimyr, [Lena, Yana, Kolyma, Magadan, Anadyr] [Point Lay, North Slope, Koyukuk, Kanuti, Selawik, Yukon, Anderson, Mackenzie] [Kent, Queen Maud, Victoria, Rasmussen] [Y-K Delta, Bristol Bay, Cook Inlet] | **0.304** | **0.263** | **0.055** | 5.5 | 0.018 |
| Geographic | [Vaygach, Taimyr, [Lena, Yana, Kolyma, Magadan, Anadyr] [Point Lay, North Slope, Koyukuk, Kanuti, Selawik, Yukon, Anderson, Mackenzie] [Kent, Queen Maud, Victoria, Rasmussen, Greenland] [Y-K Delta, Bristol Bay, Cook Inlet] | **0.307** | **0.253** | **0.072** | 7.2 | 0.007 |
| Geographic | [Greenland, Vaygach, Taimyr, Lena, Yana] [Kolyma, Magadan, Anadyr] [Point Lay, North Slope, Koyukuk, Kanuti, Selawik, Yukon, Anderson, Mackenzie] [Kent, Queen Maud, Victoria, Rasmussen] [Y-K Delta, Bristol Bay, Cook Inlet] | **0.307** | **0.250** | **0.076** | 7.6 | 0.002 |
| Geographic | [Vaygach, Taimyr, Lena, Yana] [Kolyma, Magadan, Anadyr] [Point Lay, North Slope, Koyukuk, Kanuti, Selawik, Yukon, Anderson, Mackenzie] [Kent, Queen Maud, Victoria, Rasmussen, Greenland] [Y-K Delta, Bristol Bay, Cook Inlet] | **0.309** | **0.245** | **0.085** | 8.5 | 0.002 |
| Geographic | [Greenland, Vaygach, Taimyr] [Lena, Yana, Kolyma, Magadan, Anadyr] [Point Lay, North Slope, Anderson, Mackenzie] [Koyukuk, Kanuti, Selawik, Yukon] [Kent, Queen Maud, Victoria, Rasmussen] [Y-K Delta, Bristol Bay, Cook Inlet] | **0.300** | **0.260** | **0.055** | 5.5 | 0.024 |
| Geographic | [Vaygach, Taimyr] [Lena, Yana, Kolyma, Magadan, Anadyr] [Point Lay, North Slope, Anderson, Mackenzie] [Koyukuk, Kanuti, Selawik, Yukon] [Kent, Queen Maud, Victoria, Rasmussen, Greenland] [Y-K Delta, Bristol Bay, Cook Inlet] | **0.302** | **0.249** | **0.071** | 7.1 | 0.006 |
| Geographic | [Greenland, Vaygach, Taimyr, Lena, Yana] [Kolyma, Magadan, Anadyr] [Point Lay, North Slope, Anderson, Mackenzie] [Koyukuk, Kanuti, Selawik, Yukon] [Kent, Queen Maud, Victoria, Rasmussen] [Y-K Delta, Bristol Bay, Cook Inlet] | **0.302** | **0.246** | **0.075** | 7.5 | 0.005 |
| Geographic | [Vaygach, Taimyr, Lena, Yana] [Kolyma, Magadan, Anadyr] [Point Lay, North Slope, Anderson, Mackenzie] [Koyukuk, Kanuti, Selawik, Yukon] [Kent, Queen Maud, Victoria, Rasmussen, Greenland] [Y-K Delta, Bristol Bay, Cook Inlet] | **0.304** | **0.240** | **0.083** | 8.3 | 0.002 |
| Nesting habitat | [Greenland, Vaygach, Taimyr, Lena, Yana, Kolyma, Anadyr, Point Lay, North Slope, Bristol Bay, Cook Inlet, Y-K Delta, Anderson, Mackenzie, Kent, Queen Maud, Victoria, Rasmussen][Magadan, Cook Inlet, Koyukuk, Selawik, Kanuti, Yukon] | **0.304** | **0.288** | 0.022 | 2.2 | 0.145 |
| Refugia (Ploeger 1968) | [Vaygach, Taimyr [Lena, Yana, Kolyma, Magadan, Anadyr, Point Lay, North Slope, Y-K Delta, Bristol Bay, Cook Inlet, Koyukuk, Kanuti, Selawik] [Anderson, Mackenzie, Yukon, Kent, Queen Maud, Victoria, Rasmussen] | **0.308** | **0.284** | 0.033 | 3.3 | 0.109 |
| Refugia  (Ploeger 1968) | [Vaygach, Taimyr, Lena, Yana] [Kolyma, Magadan, Anadyr, Point Lay, North Slope, Y-K Delta, Bristol Bay, Cook Inlet, Koyukuk, Kanuti, Selawik] [Anderson, Mackenzie, Yukon, Kent, Queen Maud, Victoria, Rasmussen] | **0.307** | **0.283** | 0.033 | 3.3 | 0.067 |
| Refugia  (Ploeger 1968) | [Vaygach, Taimyr, Lena, Yana] [Kolyma, Magadan, Anadyr, Point Lay, North Slope, Y-K Delta, Bristol Bay, Cook Inlet, Koyukuk, Kanuti, Selawik, Anderson, Mackenzie, Yukon] [Kent, Queen Maud, Victoria, Rasmussen] | **0.316** | **0.278** | **0.052** | 5.2 | 0.037 |

^1^Population codes given in parentheses are in reference to Figure 1 and Table 1: Bristol Bay (A), Cook Inlet (B), Y-K Delta (C), Kanuti (D), Koyukuk (E), North Slope (F), Selawik (G), Point Lay (H), Anderson (I), Mackenzie (J), Kent (K), Victoria (L), Queen Maud (M), Rasmussen (N), Yukon (O), Greenland (P), Anadyr (Q), Kolyma (R), Lena (S), Magadan (T), Taimyr (U), Vaygach (V), Yana (W).

**References**

Banks, R.C. (2011). Taxonomy of Greater White-fronted Geese (Aves: Anatidae). Proceedings of the Biological Society of Washington, 124, 226–233.

Delacour, J. (1954). The waterfowl of the world. Country Life Unlimited, London.

Hartlaub, G. (1852). Descriptions de quelques nouvelles especes d ’Oiseaux. Revue et Magasin de Zoologie Pure et Appliquée, 4 (2nd series), 3–9.

Mooij, J.H., & Zöckler, C. (2000). Reflections of the systematics, distribution, and status of Anser albifrons. Casarca, 6, 92–107.

Ploeger, P.L. (1968). Geographical differentiation in arctic Anatidae as a result of isolation during the last glacial period. Ardea, 56, 1–159.
